# *MGMT* epimutations and risk of incident cancer of the colon, glioblastoma multiforme, and diffuse large B cell lymphomas

**DOI:** 10.1186/s13148-025-01835-x

**Published:** 2025-02-20

**Authors:** Oleksii Nikolaienko, Garnet L. Anderson, Rowan T. Chlebowski, Su Yon Jung, Holly R. Harris, Stian Knappskog, Per E. Lønning

**Affiliations:** 1 Department of Clinical Science, University of Bergen, Bergen, Norway; 2 Department of Oncology, Haukeland University Hospital, Bergen, Norway; 3 Division of Public Health Sciences, Fred Hutchinson Cancer Center, Seattle, USA; 4 The Lundquist Institute, Torrance, USA; 5 Department of Epidemiology, Fielding School of Public Health, Translational Sciences Section, Jonsson Comprehensive Cancer Center, School of Nursing, University of California, Los Angeles, USA

**Keywords:** Cancer risk, *MGMT*, Epimutation, Constitutional methylation, Colon cancer, Glioblastoma, B-cell lymphoma

## Abstract

**Background:**

Constitutional *BRCA1* epimutations (promoter hypermethylation) are associated with an elevated risk of triple-negative breast cancer and high-grade serous ovarian cancer. While *MGMT* epimutations are frequent in colon cancer, glioblastoma, and B-cell lymphoma, it remains unknown whether constitutional *MGMT* epimutations are associated with risk of any of these malignancies.

**Methods:**

We designed a nested case–control study, assessing potential associations between *MGMT* epimutations in blood from healthy individuals and subsequent risk of incident cancer. The study cohort was drawn from postmenopausal women, participating in the Women’s Health Initiative (WHI) study, who had not been diagnosed with either colon cancer, glioblastoma, or B-cell lymphoma prior to study entry. The protocol included *n* = 400 women developing incident left-sided and *n* = 400 women developing right-sided colon cancer, *n* = 400 women developing diffuse large B-cell lymphomas, all matched on a 1:2 basis with cancer-free controls, and *n* = 195 women developing incident glioblastoma multiforme, matched on a 1:4 basis. All cancers were confirmed in centralized medical record review. Blood samples, collected at entry, were analyzed for *MGMT* epimutations by massive parallel sequencing. Associations between *MGMT* methylation and incident cancers were analyzed by Cox proportional hazards regression.

**Results:**

Analyzing epimutations affecting the key regulatory area of the *MGMT* promoter, the hazard ratio (HR) was 1.07 (95% CI 0.79–1.45) and 0.80 (0.59–1.08) for right- and left-sided colon cancer, respectively, 1.13 (0.78–1.64) for glioblastoma, and 1.11 (0.83–1.48) for diffuse large B-cell lymphomas. Sensitivity analyses limited to subregions of the *MGMT* promoter and to individuals with different genotypes of a functional SNP in the *MGMT* promoter (rs16906252), revealed no significant effect on HR for any of the cancer forms. Neither did we observe any effect of rs16906252 status on HR for any of the cancer forms among individuals methylated or non-methylated at the *MGMT* promoter.

**Conclusions:**

Constitutional *MGMT* promoter methylation in normal tissue is not associated with an increased risk of developing colon cancer, glioblastoma, or B-cell lymphoma.

**Supplementary Information:**

The online version contains supplementary material available at 10.1186/s13148-025-01835-x.

## Introduction

Gene silencing by epigenetic mechanisms is part of multiple normal physiological processes. Proximal gene promoter regions, located within a thousand base pairs upstream of the transcription start site, are typically characterized by a high density of CpG dinucleotides in so-called CpG islands [1]. While epigenetic gene repression involves different processes like DNA methylation and histone modifications [2], these major events in general appear in concert [3], making CpG methylation a suitable marker of epigenetic silencing of many genes.

Abnormal epigenetic regulation, coined epimutations, often detected as gene promotor hypermethylation, is frequently observed in cancer tissue and thought to play a key role in tumor evolution [4]. However, few studies have investigated constitutional epimutations associations with risk of certain cancers where gene promotor hypermethylation is more commonly seen.

Constitutional epimutations are epigenetic disturbances arising in utero, normally affecting tissues belonging to all three germ layers [5, 6]. They are classified into two major groups; primary epimutations where no associated DNA sequence variant is detected, and secondary epimutations, occurring as a consequence of a local *cis*-acting DNA sequence alteration [7, 8].

While secondary constitutional epimutations in tumor suppressor genes like *MLH1*, *BRCA1*, and *MSH2* [9–13] have been associated with elevated cancer risk, such secondary epimutations are extremely rare [14]. In contrast, low-level mosaic primary constitutional epimutations in the breast cancer 1 gene, *BRCA1,* have been detected in 5–9% of healthy females of different age, including newborns [15, 16]. Moreover, in a nested case–control ancillary study in the Women’s Health Initiative (WHI), low-level mosaic *BRCA1* promoter methylation in white blood cells (WBC) from healthy females was associated with a significantly elevated hazard ratio for incident triple-negative breast cancer (TNBC) as well as for incident high-grade serous ovarian cancer (HGSOC), both diagnosed many years after WBC DNA sampling [15]. Subsequently, we estimated that about 20% of all TNBCs may, in fact, arise from normal cells carrying constitutional *BRCA1* epimutations [16]. These findings raised the question of whether low-level mosaic constitutional epimutations in other tumor suppressor genes may be an underlying cause of cancer in other organs as well.

A potential candidate gene for such a role is O^6^-methylguanine-DNA methyltransferase, *MGMT*, pivotal to O^6^-methylguanine detoxification [17]. *MGMT* promoter methylation is frequently observed across many tumor forms, and low-level mosaic *MGMT* methylation has been detected in WBC of > 10% of newborns and adult women [18, 19] (Nikolaienko et al.; unpublished results). Moreover, *MGMT* epimutations are strongly linked to the alternative allele of rs16906252C > T, located within a *cis*-acting enhancer element in exon 1, and conflicting data have suggested the alternative allele itself may play a role in pathogenesis [20–26].

Here, we assessed the potential association between antecedent WBC *MGMT* methylation state in healthy people and incident colon cancer (CC), glioblastoma (GB), and diffuse large B-cell lymphoma (DLBCL), three tumor forms known to have *MGMT* epimutations affecting 20–40% of all tumors [17, 27–30].

## Methods

### Study population and design

The Women’s Health Initiative Study (WHI) was initiated in 1993**.** In WHI [31, 32], normal blood DNA was obtained from > 160,000 American women aged 50–79 years at study entry, after which participants were followed over 2 decades for incident diseases, including cancers, with detailed information, including histopathological confirmation of diagnosis. Demographic characteristics, family history, and reproductive and medical history were collected by self-administered questionnaires at entry, and race and ethnicity were self-reported against fixed categories.

DNA was obtained from WBC samples collected at enrollment. Samples were collected after at least 12 h of fasting according to pre-specified standard procedures and shipped on dry ice and stored at − 80 °C at Fisher Bioservices (Rockville, Maryland).

Clinical outcomes were ascertained annually from enrollment in the observational study and every 6 months for clinical trial participants during the 8.5-years intervention period and annually thereafter. Self-reported cancers were confirmed by medical record review at the clinical centers by trained physician adjudicators and, subsequently, by a final confirmation at the clinical coordinating center.

Study protocols were approved at all clinical centers, and participants provided written informed consent. In addition, the current study was approved by the Regional Ethical Committee of the Western Norwegian Health Region.

The study was conducted as three nested case–control studies (study protocol in Additional file 1, with addendum in Additional file 2). Full details of the statistical power analyses are given in the study protocol. Because *MGMT* epimutations are significantly associated with the alternative allele of rs16906252C > T, and conflicting data have suggested an independent pathogenic role of the alternative allele [20–26], the study was powered to assess the role of epimutations and rs16906252 status separately. Thus, including 400 cases of cancer patients matched in a 1:2 nested case–control design allowed evaluation of a HR of 2 for *MGMT* epimutations and the alternative rs16906252 allele separately with a statistical power > 0.9. This design was applied to left- and right-sided colon cancer separately and to DLBCL. As the number of cases for each group exceeded n = 600, cases were randomly selected. For glioblastoma, where the number of cases was limited to 195, an increased number of controls was required. Here, a HR of 2.0 may be determined separately for *MGMT* and the rs16906252 alternative allele with a power > 0.8 using a 1:4 matched design.

In brief, analyses were powered for successful *MGMT* promoter methylation analysis from cases with incident left- (*n* = 400) and right-sided (*n* = 400) colon cancer, incident DLBCL (*n* = 400), and incident glioblastomas (*n* = 195), with cancer-free controls matched on a 1:2 basis except for glioblastomas (1:4; Fig. 1). Matching criteria for the controls were age at entry, race and ethnicity, hormone therapy use, smoking, and DNA extraction method. Women reporting a history of either cancer form at baseline were excluded. Additional exclusion criteria were lack of follow-up, lack of smoking history or DNA sample missing, and specific to glioblastoma and DLBCL controls: history of brain, leukemia, Hodgkin’s lymphoma, or non-Hodgkin’s lymphoma cancer reported at baseline or adjudicated during follow-up. Exclusions specific to colon cancer controls: history of colorectal cancer, ulcerative colitis or Crohn's disease at baseline, or colorectal cancer during follow-up. To limit the required sample number, some controls were used for comparison for more than one cancer form (Additional file 3). Controls were first drawn for the glioblastoma comparison, followed by DLBCL and finally colon cancer. Further, controls were required to be alive and disease-free at the time of the case diagnosis (for details, see Additional file 3).

**Fig. 1:**
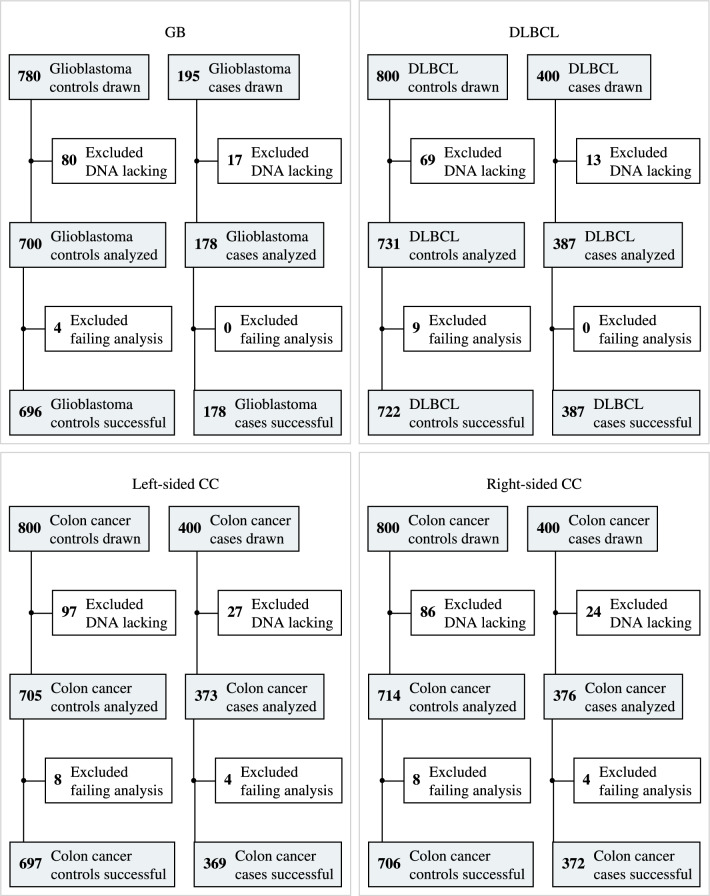
Flowchart depicting samples drawn and successfully analyzed from patients and controls. Out of the 2,410 controls, 440 served as controls for both GB and DLBCL

The study was conducted in accordance with the STROBE recommendations (https://www.strobe-statement.org/).

### *MGMT* promoter methylation analysis

**Fig. 2:**
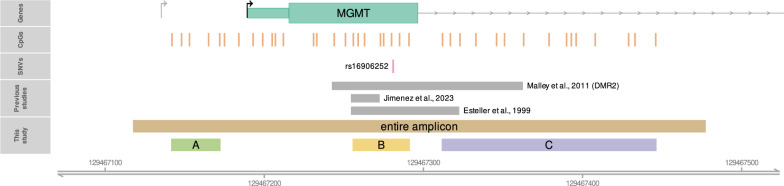
The genomic structure of the *MGMT* promoter region. Upward arrows with rightward tips show transcription start sites according to Harris et al. [40] (lightgray) and GRCh38.p14 genome assembly annotation (black). The first *MGMT* exon is depicted by green rectangles, where the thin left part represents 5’UTR. The covered CpGs are indicated by orange vertical lines. The single nucleotide variant rs16906252 is indicated by a pink vertical line. Regions commonly analyses in other studies [24, 36, 41] are indicated by gray rectangles. The amplicon and the three analyzed regions in the present study are indicated at the bottom by brown, light green, yellow, and purple rectangles

Studies assessing cancer risk and impact of *MGMT* promoter status on cancer biology have analyzed different upstream *MGMT* regions, with a majority focusing on a region of exon 1 [24, 33–38]. Our assay covered a larger region (GRCh38 chr10:129467118–129467477), including the 3’ part of the *MGMT* promoter, the entire exon 1, and the 5’ part of intron 1 (Fig. 2). We initially planned to average methylation status across the entire region covered (Protocol in Additional file 1). Upon completing the methylation analysis of the full sample set and performing initial quality control and methylation calling blinded to case–control sample status, we discovered a significant difference in methylation frequency between subregions of the covered region. Dividing the region into subregions A, B, and C (Additional file 2) we found methylation being much less abundant in area A as compared to B and C. We therefore revised the protocol prior to unblinding of case–control status (Additional file 2) and defined region B, i.e., an area in *MGMT* exon 1 and overlapping with the area assessed in most previous publications, to be the target for our primary hazard ratio analyses, while hazard ratios related to regions A and C, as well as to the entire amplicon area, were defined as secondary analyses.

A full description of the analytical method is given in Methods section of Additional file 4. In brief, WBC-based genomic DNA samples were bisulfite converted and the *MGMT* promoter area was amplified, indexed, and sequenced to an ultrahigh depth of coverage (> 30.000×) using the Illumina MiSeq System.

All samples were analyzed blinded to case–control status. Reads were mapped/aligned to the GRCh38 reference genome using Illumina DRAGEN Bio-IT Platform, and the methylation was assessed using epialleleR R package [39]. According to the study protocol (Additional files 1 and 2), an epimutation was defined as a methylation pattern per allele with at least 75% of CpGs being informative (not missing methylation status), and more than a half of the informative ones being methylated, and an epimutation-positive sample was defined as a sample with the coverage of at least 10,000× and an epimutation frequency (Variant Epiallele Frequency, VEF) of at least 1/10,000.

### Determining allele specificity of methylation

The region covered by sequencing contains a frequent single nucleotide polymorphism (SNP), rs16906252C > T (Fig. 2). By analyzing methylation in sequencing reads covering this SNP, we assessed the potential allele specificity of methylation in individuals heterozygous for the SNP.

### Statistical analysis

The aim of the study was to assess potential associations of *MGMT* methylation and rs16906252C > T status independently with risk of incident cancer of the colon, DLBCL, or glioblastomas each. This was done by estimating hazard ratios (HR) and 95% confidence intervals (CI) using Cox proportional hazards regression in matched case–control groups, including age, race, previous hormone usage, smoking, and DNA extraction method as covariates. In addition, we performed hypothesis-generating supportive subgroup analyses.

## Results

### Participant demographics and methylation characteristics

The demographics and consort diagram for cancer cases and cancer-free controls are presented in Table 1 and Fig. 1. Demographic parameters were well balanced between cancer cases and their controls, due to the matching design.

**Table 1:** …

	GB cases (*N* = 178)	DLBCL cases (*N* = 387)	Left-sided CC cases (*N* = 373)	Right-sided CC cases (*N* = 376)	Controls (*N* = 2410)
*Age*					
Mean (SD)	63.2 (6.81)	64.2 (6.92)	64.3 (6.99)	65.3 (6.51)	64.0 (6.83)
Median [Min, Max]	63.0 [50.0, 79.0]	64.0 [50.0, 79.0]	64.0 [50.0, 79.0]	66.0 [50.0, 79.0]	64.0 [50.0, 79.0]
IQR [Q1, Q3]	10.0 [58.0, 68.0]	11.0 [59.0, 70.0]	11.0 [59.0, 70.0]	9.00 [61.0, 70.0]	10.0 [59.0, 69.0]
*Ethnicity*					
Not Hispanic or Latino	170 (95.5%)	370 (95.6%)	355 (95.2%)	363 (96.5%)	2307 (95.7%)
Hispanic or Latino	5 (2.8%)	13 (3.4%)	12 (3.2%)	10 (2.7%)	94 (3.9%)
Unknown or not reported	3 (1.7%)	4 (1.0%)	6 (1.6%)	3 (0.8%)	9 (0.4%)
*Race*					
American Indian or Alaskan Native	0 (0%)	1 (0.3%)	0 (0%)	4 (1.1%)	6 (0.2%)
Asian	2 (1.1%)	7 (1.8%)	7 (1.9%)	7 (1.9%)	48 (2.0%)
Native Hawaiian or Other Pacific Islander	0 (0%)	0 (0%)	0 (0%)	0 (0%)	2 (0.1%)
Black or African-American	4 (2.2%)	8 (2.1%)	38 (10.2%)	32 (8.5%)	165 (6.8%)
White	169 (94.9%)	364 (94.1%)	314 (84.2%)	321 (85.4%)	2127 (88.3%)
More than one race	0 (0%)	3 (0.8%)	7 (1.9%)	6 (1.6%)	32 (1.3%)
Unknown or not reported	3 (1.7%)	4 (1.0%)	7 (1.9%)	6 (1.6%)	30 (1.2%)
*Years from DNA sampling to diagnosis*					
Mean (SD)	9.72 (7.11)	12.3 (6.73)	8.89 (6.48)	11.8 (6.62)	NA
Median [Min, Max]	9.00 [0, 25.0]	12.0 [0, 28.0]	8.00 [0, 28.0]	12.0 [1.00, 27.0]	NA
IQR [Q1, Q3]	11.8 [3.00, 14.8]	10.0 [7.00, 17.0]	9.00 [4.00, 13.0]	11.0 [6.00, 17.0]	NA
Missing	0 (0%)	0 (0%)	0 (0%)	0 (0%)	2410 (100%)
*Hormone use*					
Never used	76 (42.7%)	165 (42.6%)	203 (54.4%)	193 (51.3%)	1172 (48.6%)
Unopposed estrogen	45 (25.3%)	118 (30.5%)	94 (25.2%)	103 (27.4%)	698 (29.0%)
Estrogen + progesterone	57 (32.0%)	104 (26.9%)	76 (20.4%)	80 (21.3%)	540 (22.4%)
*Smoking status*					
Never smoked	84 (47.2%)	207 (53.5%)	187 (50.1%)	176 (46.8%)	1205 (50.0%)
Past smoker	82 (46.1%)	167 (43.2%)	162 (43.4%)	171 (45.5%)	1046 (43.4%)
Current smoker	12 (6.7%)	13 (3.4%)	24 (6.4%)	29 (7.7%)	159 (6.6%)

*MGMT* promoter methylation frequency varied significantly between subregions of the area covered by our assay. Methylation frequency was low in region A (located upstream of *MGMT* exon 1) as compared to B (located within exon 1) and C (located in intron 1). The epimutation frequencies in these three subregions among controls were 9.2%, 26.8%, and 27.1%, respectively. A similar difference was seen among cancer cases (A: 8.6%, B: 26.7%, and C: 27.1%, for all cases merged) and for each cancer types separately (Additional file 4). The details on the distribution of methylation within alleles are given in Additional file 2 and Additional file 4, with individual details in Additional file 5.

### *MGMT* methylation and risk of colon cancer, DLBCL, or glioblastoma

The median follow-up time from entry to diagnosis, for cases diagnosed with cancer of the left- or right-sided colon, glioblastoma, and DLBCL was 8, 12, 9, and 12 years, respectively.

**Fig. 3:**
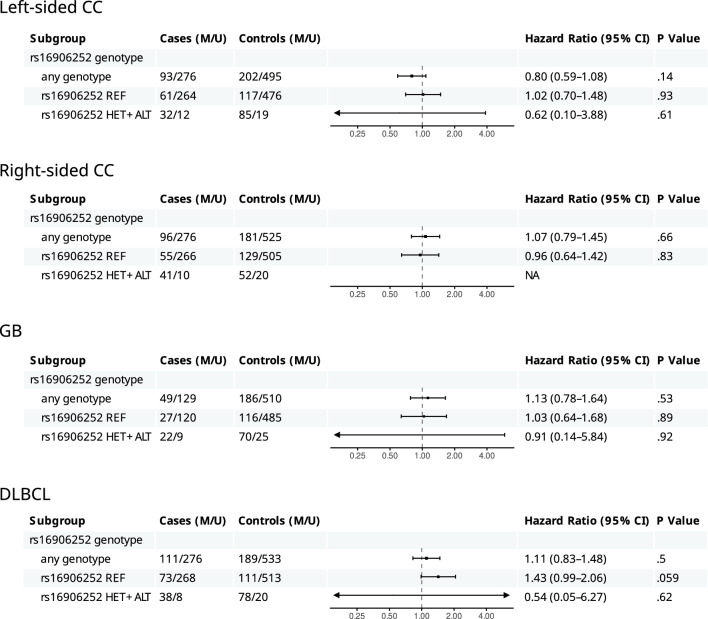
Hazard ratios for left- and right-sided colon cancer, GB, and DLBCL associated with the presence of methylated *MGMT* alleles in the overall cohorts and subgroups based on rs16906252 status. M and U are samples with methylated and unmethylated *MGMT* promoter, respectively. REF, HET and ALT are CC, CT and TT genotypes of rs16906252, respectively

*MGMT* methylation in the subregion B, selected as the primary endpoint, was not associated with an elevated HR for any of the cancer types analyzed (Fig. 3). The lack of association was also observed when data were stratified according to rs16906252 genotype.

Assessing alternative regions (regions A and C as well as the entire area covered by our assay), a similar lack of association was mainly observed (Additional file 4, Figs. S11–S15). In some isolated comparisons, epimutations were seemingly associated with the risk of particular cancers (e.g., DLBCL by epimutations within area C). However, most of these associations become nonsignificant upon correction for multiple comparisons. Further, the largely overlapping shapes of epimutation frequency density distributions for cases and controls (Additional file 4, Figs. S5–S10) confirm that these seemingly significant observations are due to chance only.

### *MGMT* rs16906252 status and risk of colon cancer, glioblastoma, and DLBCL

**Fig. 4:**
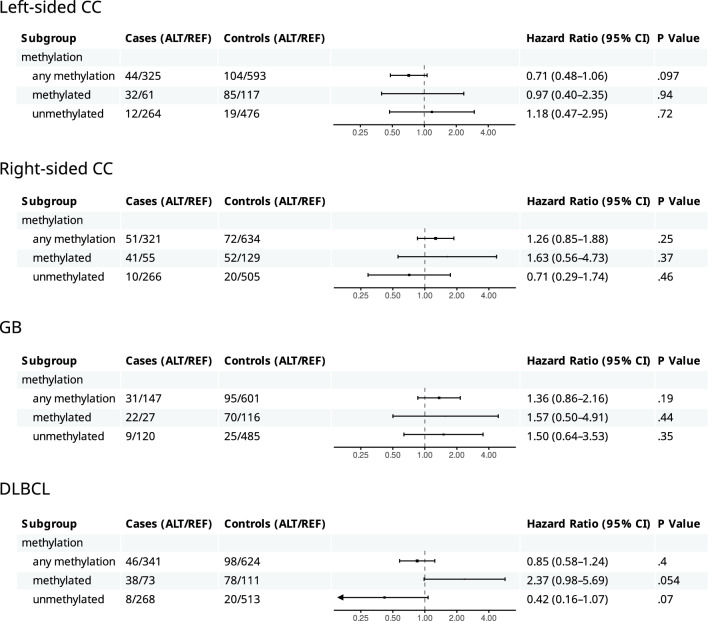
Hazard ratios for left- and right-sided CC, GB, and DLBCL associated with rs16906252 status in the overall cohorts and subgroups based on *MGMT* methylation status. REF and ALT are CC and CT+TT genotypes of rs16906252, respectively

The HR for each cancer type with respect to the rs16906252 variant allele (hetero- and homozygotes combined) for segment B of the promoter is presented in Fig. 4, with additional sub-analysis in Additional file 4, Figs. S16-S20. Mirroring the findings for *MGMT* methylation, we found no association between *MGMT* rs16906252 status and risk of any of the three cancer types.

## Discussion

The incidence rates for most cancers have increased significantly in all Western countries over the last decades. While general opinion relates this increase to lifestyle and environmental influences, like food ingestion and different types of exposure to polluting agents, in general, we lack direct molecular evidence linking increased cancer risk to defined factors. Further, immigration studies revealing a strong impact on cancer risk in second-generation immigrants [42–45] suggest that risk factors may be related to early-life events.

While our knowledge regarding the potential role of epimutations to cancer risk and evolution remains limited, pollution and several carcinogenic agents in our environment are known to induce epigenetic disturbances [46–49]. Further, even if epigenetic events are most commonly considered to evolve during lifetime [50] and cancer to evolve as a multistep process including sequential alterations over time [51], early-life epigenetic events may be of particular interest as a factor changing the general human cancer risk without requiring long-term selection of genetic variants. Most constitutional epimutations, arising in utero, that have been reported so far, are secondary, linked to inherited genetic variants and their frequency in the population is very low [5]. However, our recent findings of low-level mosaic primary epimutations in the *BRCA1* gene, affecting 5–9% of all females and being associated with an elevated risk of both triple-negative breast cancer and high-grade serous ovarian cancer [15, 16], focused on early-life events affecting a large fraction of the population, resulting in increased cancer risk. Thus, most likely, similar primary epimutations arising on a non-genetic background may be risk factors to other cancer forms as well.

Tumor suppressor genes like *BRCA1*, *BRCA2*, and mismatch repair genes are involved in DNA repair following genomic damage. Germline pathogenic variants in these genes are associated with highly increased cancer risk. While germline pathogenic variants affecting *MGMT* have not been identified, the gene plays a pivotal role in DNA damage repair [17], and epimutations presenting as promoter hypermethylation in tumors have been found associated with sensitivity toward alkylating agents like temozolomide and dacarbazine [17, 52].

While colon cancers are known to harbor methylation in many genes in addition to the *MGMT* [53], *MGMT* is found frequently epimutated in WBC, indicating a constitutional origin. The fact that *MGMT* methylation has been detected in colon adenomas as well as normal colon mucosa located 10 cm from the tumor borders [54–56], suggests the presence of normal tissue epimutations which, potentially, may act as cancer precursors. While a potential role for *MGMT* constitutional epimutations to cancer risk has not been prospectively evaluated, *MGMT* epimutations are strongly associated with the alternative allele of rs16906252. Thus, in a large study of germline genotypes (WBC) including a validation cohort, Kuroiwa-Trzmielina and colleagues found the rs16906252 alternative T-allele to be associated with an odds ratio (OR) of 3–4 for developing *MGMT* promoter–methylated colorectal cancer. Surprisingly, they also observed a significant reduced risk of developing *MGMT* methylation–negative colorectal cancers [26]. In that study, samples for formal assessment of HR related to *MGMT* WBC methylation were not available; thus, a potential direct association between normal and cancer tissue *MGMT* epimutations could not be assessed. Apart from a small study indicating an association between the rs16906252 alternative T-allele and glioblastoma risk [20], the potential risk for different cancers related to rs16906252 status per se, has not been addressed.

To evaluate a potential relationship of *MGMT* constitutional methylation and the rs16906252 genotype on cancer risk, we took advantage of the prospective Women’s Health Initiative study, designing a nested case–control study to evaluate the risk of three different major cancer forms. The cancer forms were selected on the basis of high fractions of tumors with *MGMT* methylation. We evaluated *MGMT* epimutations across three subregions covering *MGMT* promoter and flanking regulatory areas. The study is statistically well powered to detect a potential increase in HR of each individual cancer, both in respect of *MGMT* epimutations and rs16906252 status. Taken together, our findings are negative with respect to all three tumor forms.

Previously, Kuroiwa-Trzmielina et al. [26] showed that individuals carrying the rs16906252 T-allele have an increased risk of *MGMT*-methylated tumors but reduced risk of non-methylated ones. Together with our data, this raises the question of whether *MGMT* methylation may be a secondary event during early carcinogenesis, directing a different tumor biology.

The study has several limitations. The WHI cohort includes women only. While we do not have any reason to believe that constitutional *MGMT* methylation may act differently with respect to gender-related risk for the cancer forms investigated, the effect in males has not been formally investigated.

All WHI participants were postmenopausal at time of entry. The median age was 64 years with 24% ≥ 70 years of age at entry. Thus, a potential influence on cancer risk at young age may be undetected.

Information about *MGMT* germline pathogenic variant status was not available for the current study participants. While such variants, in theory, may affect the cancer risk of individuals harboring them, to the best of our knowledge, germline pathogenic variants in the *MGMT* gene have never been reported; in case they exist, they must be rare.

For any case–control study assessing cancer risk like the present, the findings should ideally be validated in independent larger cohorts. There are, however, few population-based cohorts, like WHI, enrolling numbers of participants that may provide adequate statistical power. The fact that we see the same results across three different cancer types, all characterized by frequent somatic *MGMT* methylation, at least provides indirect validation between cancer types.

## Conclusion

We found no association between constitutional normal tissue (WBC) methylation of the *MGMT* promoter and subsequent risk of colon cancer, glioblastoma multiforme, or B-cell lymphoma.

## Supplementary Information

Additional file1: Study protocol

Additional file2: Analytical addendum

Additional file3: Sample selection protocol WHI

Additional file4: Additional methods and results

Additional file5: Sequencing coverage

## Data Availability

No datasets were generated or analyzed during the current study.
