# Supplementary material for: MGMT epimutations and risk of incident cancer of the colon, glioblastoma multiforme, and diffuse large B cell lymphomas
Source: Clin Epigenetics. 2025 Feb 20;17:28. doi: 10.1186/s13148-025-01835-x (PMC11841191; doi:10.1186/s13148-025-01835-x)
Supplement: Supplementary file 1 — Additional file1: Study protocol [file 13148_2025_1835_MOESM1_ESM.pdf]

Protocol: **Exploring *MGMT* constitutional methylation and the *MGMT* SNP rs16906252 as risk factors for cancer coli, glioblastomas and diffuse large B-cell lymphoma in the WHI study**

August 17, 2022

Proposal by: **Per E Lønning**, MD and PhD, Professor and Consultant Oncologist, Head Breast Cancer Section and the Breast Cancer Translational Research Group, Department of Clinical Science and Department of Oncology, Mohn Cancer Research Laboratory, University of Bergen and Haukeland University Hospital

Stian Knappskog, PhD, Professor, Breast Cancer Translational Research Group, Department of Clinical Science and Department of Oncology, Mohn Cancer Research Laboratory, University of Bergen and Haukeland University Hospital

WHI-investigator: **Rowan Chlebowski**, MD and PhD, Professor UCLA

Background.

The incidence rates for most cancers have increased significantly in all Western countries over the last decades. Comparing age-adjusted incidence rates for some major cancer forms during the time period of 1959-63 versus 2014-18 in Norway, cancer of the colon and rectum increased by about 150% in both sexes, testicular cancers by 200%,, brain tumours by 150%, while breast and prostate cancer both increased by more than 100% (Norwegian Cancer Registry; https://www.kreftregisteret.no/Registrene/Kreft_i_Norge/). Within the same time-span, there has been a 9-fold increase in melanoma incidence and a 10-fold increase in lung cancer incidence in females. These increased incidences are likely due to changes in UV-light exposition and smoking habits. In addition, a reduction of 85% in stomach cancer is likely related to better-preserved food. Importantly, however, for most major cancer forms we lack an explanation for the increased incidence. While factors like an increase in obesity rates have been linked to cancer incidence, this presents by no mean the full explanation. Notably, immigration studies from different countries have revealed a strong impact on cancer risk in second-generation immigrants (1-4), indicating environmental influence to be of key importance.

Occupational exposure to toxic agents (asbestos, benzene and many environmental pollutants) are known to increase cancer risk. However, while a general opinion is that increased risk of many cancers in the western population may be due to environmental influences like food ingestion and different types of exposure to polluting agents, in general we lack direct evidence linking enhanced cancer risk to defined factors. Moreover, we have limited knowledge regarding molecular mechanisms by which polluting agents may induce distinct cancer forms. While defined toxic agents (like cigarette smoking and UV exposure) are associated with distinct genomic signatures, reflecting physical or chemical damage on DNA in certain cancer forms (5), as for most cancer forms defined mutation signatures related to exogenous agents have not been identified.

Genetic silencing by epigenetic regulation is part of multiple normal physiological processes and plays a pivotal role during embryogenesis. Gene promoter regions, in general

located within a thousand base pairs upstream of the gene, are typically characterized by a high density of CpG dinucleotides into so-called CpG islands (6). Epigenetic regulation involves at least two different processes, CpG methylation, and histone demethylations (7). These two events in general appear in concert (8), making CpG methylation a suitable marker of epigenetic silencing of many genes. Abnormal epigenetic regulation is coined epimutations and may occur *in utero*, so-called **constitutional epimutations**, or in different tissues later in life as **somatic epimutations**. The fact that epigenetic regulation is influenced by genetic but also environmental factors including pollutants (9-14) makes **epimutations** strong candidates linking environmental agents to cancer risk.

Constitutional epimutations presents epigenetic disturbances arising *in utero*, normally affecting tissues belonging to all three germ layers (15, 16). **Constitutional methylations** are classified into two major groups; **primary epimutations** where no associated DNA sequence variant is detected, and **secondary epimutations,** occurring as a consequence of a local *cis*-acting DNA alteration (17, 18). While gene silencing through somatic epimutations are frequently observed in cancer tissue (19), despite some preliminary reports (evidence summarized in (20)), very few studies have investigated constitutional epimutations as potential underlying causes in carcinogenesis. Carcinogenesis evolve over time, including early life events (21) and for certain cancer forms, indirect evidence points toward prenatal events to be of importance for later carcinogenesis (22).

While secondary constitutional epimutations in tumor suppressor genes like *MLH1*, *BRCA1* and *MSH2* (11, 23-26) have been associated with elevated cancer risk, such secondary epimutations are extremely rare (20). Notably, secondary epimutations in general are easily detected due to the fact that they typically affect approximately 50% of the alleles. In contrast, while primary epimutations seems to occur more frequently, they may easily be overlooked due to a low-mosaic expression (27).

Evidence supporting mosaic methylation may be a cancer risk factor.

In a previous study, enrolling more than 4.000 patients diagnosed with high-grade serous ovarian cancer (HGSOC) and controls (27), we found low-mosaic *BRCA1* WBC methylation in 4-7% of healthy newborns (umbilical blood) as well as adults. In adult women, WBC methylation was associated with a 2.2 – 2.9 OR for HGSOC. A potential bias of that study, like all the other studies in this area (27-30), was the collection of blood samples after diagnosis.

To avoid the bias of tumor-related effects on WBC methylation, we recently assessed *BRCA1* methylation status in WBC DNA collected from >5.000 women in the American Women Health Initiative (WHI) study (31) out of whom about 660 later developed triple negative breast cancer (TNBC) and 550 high-grade serous ovarian cancer (HGSOC), in a case-control design study. The results showed a significant elevated OR both for incidental TNBC and HGSOC, even in sub analyses limited to women developing their cancers >5 years after WBC sampling (paper submitted). The results for TNBC is revealed below:

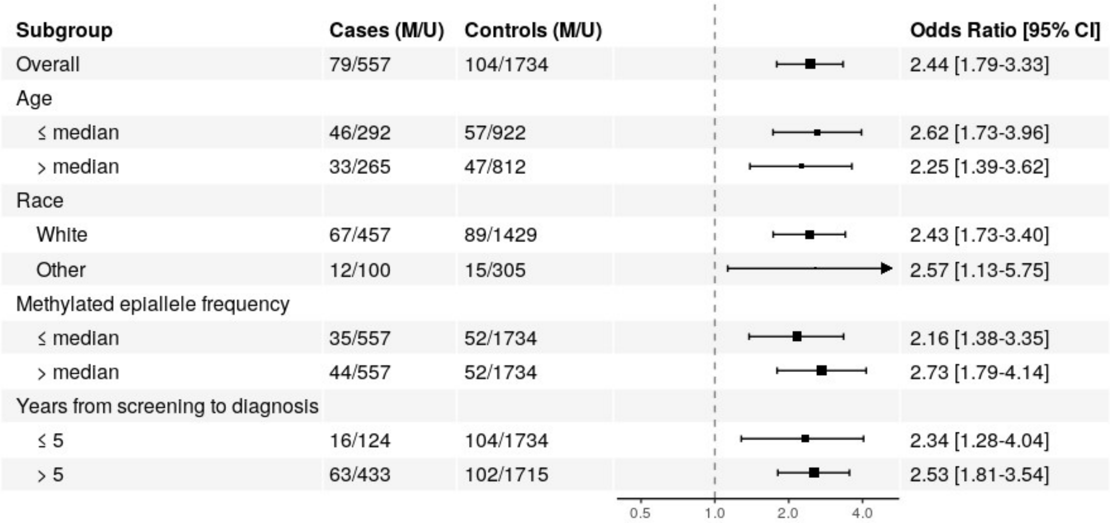

This, for the first time, confirms normal tissue methylation to be predictive of subsequent cancer development in a prospective setting. Moreover, *BRCA1* promoter methylation occurred as a monoallelic event, in affected individuals, consistent with early origin of the methylation, followed by clonal expansion.

Finally, the fact that mosaic germline mutations may confer risk of cancers as well as other medical conditions (32-39) supports the hypothesis that mosaic epimutations may confer risk of disease as well.

Study Approach.

One approach searching for novel cancer risk factors is to assess alternative inactivation mechanisms for genes in which germline mutations are strongly associated with cancer. **We have already applied this approach successfully by identifying methylation of the *BRCA1* gene as a risk factor for breast and ovarian cancer (see below). There are other candidate for which epimutations may be a potential cancer initiating factor.** Lynch Syndrome is an autosomal dominant genetic condition caused by germline mutations in genes regulating DNA mismatch repair defects (*MLH1*, *MSH2*, *MSH6*, *PMS1* or *PMS2*) (40). The syndrome is characterized by an elevated risk of colorectal and endometrial cancer as well as several other cancer forms, and the tumors characteristically express so-called microsatellite instability; MSI) (41-44) While the Lynch syndrome accounts for only 2-3% of all colon cancers (45, 46), up to 15% of all colon cancers are defined as MSI+ and the majority of these MSI+ colon cancers carries methylation of the *MLH1* promoter (47-52). This, together with the finding of *MLH1* methylation in adjacent normal colon tissue (51) indicates *MLH1* methylation to be an early event in carcinogenesis. In endometrial cancers, about 30% are found to harbor methylation of the *MLH1* gene promoter (53, 54). While secondary *MLH1* epimutations and high-VAF *MLH1* epimutations without genomic defects have been reported in association with MSI+ colon cancer, (23-25, 55-66), to this end a total of less than 50 individuals have been reported in the literature. In contrast, while low-level mosaic WBC methylation of *MLH1* in patients with colorectal cancer has been reported (56, 63), the potential implications of such low-level methylation to colorectal cancer risk has not been formally assessed. While constitutional methylation of *MSH2* has been described in a few families (11, 66, 67), these epimutations all are secondary due to a deletion in the *TACSTD1* gene located upstream of *MSH2*.

However, there is evidence suggesting normal colon (and probably other tissues as well) methylation of the *MLH1* gene to be an acquired event during lifetime. Many colon cancers are known to carry promotor methylation of different genes, partly in concert, in the so-called CIMP phenotype, and much of this methylation arise in an age-dependent manner (68-70). The

time-point when *MLH1* methylation occurs remains unsettled. Most importantly, studying low-level mosaic methylation in WBC from newborns, we found *MLH1* promoter methylation to occur in less than 0.5% of individuals only. While this finding does not exclude an organ-specific embryonic origin of methylation of normal colon cells, such an event must have happened after separation of the primordial layers (colon = endodermal derived), excluding a model using WBC methylation as a potential surrogate marker for constitutional methylation of endodermal derived tissue.

A second approach is to look for tumor suppressor genes frequently methylated across a panel of cancer forms, like the ***MGMT*** gene (71). *MGMT* is pivotal to O^6-methylguanine detoxification. The importance of this mechanism is illustrated by loss of *MGMT* expression being associated with sensitivity towards alkylating chemotherapeutics (72). *MGMT* is downregulated by promoter methylation in various types of cancers like colon cance glioblastomas and diffuse large B-bell lymphomas, affecting 20-40% of all tumors in all three groups (72-78) .

Most importantly, findings by us (>1,000 newborns studied) and others (79) have detected mosaic *MGMT* promoter methylation in white blood cells (WBC) in about 15% of newborns. Thus, constitutional methylation of *MGMT*, similar to *BRCA1*, is an embryonic event affecting a significant number of individuals. **Importantly, in case we may confirm an association between *MGMT* methylation and cancer risk, this may indicate a key role of specific genotoxic events in the carcinogenic process of the actual tumors** (71).

For most genes, we lack detailed knowledge about exactly which CpG's in the promoter area that needs methylation to cause transcription silencing. Moreover, we lack knowledge whether promotor methylation always develops as a "one-step-process" (as outlined in model A and B in Figure 1) or may develop stepwise (model C in Figure 1) for which there is some indirect evidence (80-83). Notably, while in our study of the *BRCA1* gene, we found a methylation pattern consistent with model A, this by no means excludes the possibility of different patterns among other genes subject to constitutional methylation. In the present study of *MGMT* methylation, in addition to evaluating the cancer hazard ratios (HR) related to elevated methylation status defined by Variant Epiallele Frequency (VEF; as in our previous study), we will conduct extensive formal assessment of these models based on data generated by Next-Generation Sequencing (NGS) technology.

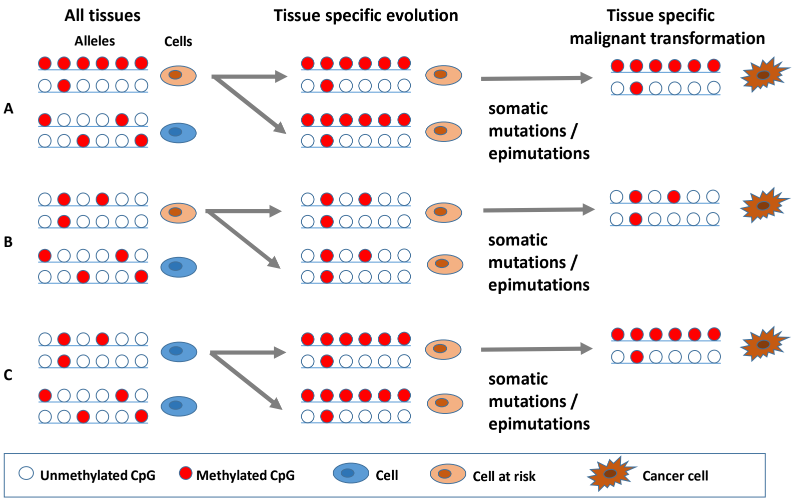

Figure 1. Examples of different models for organ-specific effects of constitutional methylation. A: **Clonal expansion**, B: **Critical CpG methylation:** some CpGs are critical to gene silencing, C: **Dynamic methylation**; promotor methylation may evolve as a step-wise process

MGMT methylation in cancer.

MGMT is a tumor suppressor, pivotal to O^6-methylguanine detoxification, downregulated by promoter methylation in various types of cancers like colon cancer and glioblastomas affecting 30-40% of all tumors in both groups. Preliminary findings by us and others (79) have detected MGMT promoter methylation in WBC in about 15% of newborns. About 30-40% of all colon cancers but also colon polyps, acting as pre-cancer lesions, as well as other cancer forms like glioblastomas reveal hypermethylation of the *MGMT* promoter (72-78) . Notably, *MGMT* methylation has been associated with sensitivity towards alkylating chemotherapeutics (72, 84, 85). While germline pathogenic mutations in the *MGMT* gene have not been reported, the T-allele of the SNP rs16906252, located close to the transcription start site in the first exon of *MGMT*, has been associated with elevated promoter methylation across a panel of solid malignancies (73, 86-90). *MGMT* methylation however has been detected in normal colon mucosa located 10 cm from the tumor borders (91), questioning the possibility of tissue-specific methylation. There may be several explanations for this finding, including local clonal expansion of constitutional methylated cells as a pre-cancer stage. Thus, we (unpubl observations) and others (92) have detected mosaic *MGMT* methylation in WBC in > 15% of newborns.

For the present project, we selected colon cancers and glioblastomas to assess potential impact of *MGMT* constitutional methylation status on cancer HR. In addition, we searched the literature for a potential third cancer type suitable for this project. The requirements were a) a cancer form for which a suitable fraction of tumors are found methylated at the *MGMT* promoter, and b) the number of participants in the WHI diagnosed with this cancer form should allow for statistical testing of HR based on upfront power estimates. Based on these criteria, we selected diffuse large B-cell lymphomas, a tumor form for which between 20 and 40% of the tumors have been reported methylated for the *MGMT* gene (88, 93-96). Regarding numbers, >500 cases have been diagnosed in the WHI data-set.

Assessing *MGMT* epimutations as a potential cancer risk factor would address several important questions. First, the fact that it specifically detoxifies O^6-methylguanine would link *MGMT* inactivation to a specific mechanism of carcinogenesis (71). Next, it may present the first gene acting as a cancer risk factor through constitutional epimutations for which germline mutations have not been detected. Third, assessing the SNP variant in concert with epimutation status, *MGMT* could be a gene for which both primary and secondary epimutations may be assessed separately and/or in concert as cancer risk factors. Forth, and most important: the three tumours selected represent a spectrum of different tumor forms in respect to biology and hypothesis on carcinogenesis in general. Colon cancer has been strongly associated with diet, indicating local exposure to the epithelial lining could play an important role. Thus, an interaction between *MGMT* deactivation in epithelial lining cells and carcinogen exposure may be hypothesized. As for glioblastomas and high-grade lymphomas, potential risk factors remain unknown. Finally, the fact that these three cancers originate from tissue derived from all the three different germ layers (colon: endoderm, glioblastomas; ectoderm, and lymphomas: mesoderm) make these three cancer forms in concert of interest, considering the fact that constitutional methylations in general may affect all three germ-cell layers.

Current study.

Here, we will confirm constitutional primary epimutations of *MGMT* to be a cancer risk factor by evaluating WBC *MGMT* methylation as risk factor for colorectal cancer, glioblastomas and high-grade lymphomas. Data generated will be used also to model the mechanism(s) of CpG methylation building up across the promotor area, evaluating the different models outlined in Figure 1.

Specific aims.

1. **We will assess the HR for right-sided and left-sided colon cancer separately related to WBC *MGMT* methylation status using a case-control designed study**
2. **We will assess the HR for glioblastoma multiforme related to WBC *MGMT* methylation status using a case-control designed study**
3. **We will assess the HR for high-grade diffuse B-cell lymphomas related to WBC *MGMT* methylation status using a case-control designed study**

Significance of findings.

The findings of this study may have three major implications. First, it may add pivotal evidence to our understanding of the mechanism of carcinogenesis. In our previous study we found primary constitutional mosaic epimutations in the *BRCA1* gene to be associated with subsequent risk of triple-negative breast cancer and high-grade serous ovarian cancer. These findings raises the pivotal question of whether cancer risk related to primary epimutations may be limited to *BRCA1* as a single gene, or similar mechanisms may affect other genes enhancing the risk of other cancer types. The answer to this question is of huge importance to our understanding of the potential role of intrauterine events to cancer risk.

Second, in case we detect normal tissue epimutations in *MGMT* to be associated with incidental cancer risk, that will add important information to our understanding of cancer-initiating events in general. The fact that germline mutations in the *TP53* gene in the Li-Fraumeni syndrome, *BRCA1/2* germline in breast / ovarian cancer families and *MLH1*/*MSH2* mutations in families with colon cancer are associated with high tumor risk confirms mutations in these genes to be initial triggering events in the carcinogenic process among these patients. On the other hand, *TP53* mutations are known also to arise as somatic events later during cancer evolution (97, 98), and epimutations in general are considered of somatic origin. Thus, information revealing that epimutaitons are not always late/somatic but occur at the embryonic stage and confer cancer risk later in life, will provide important information for a better understanding of the role of key driving mutations and epimutations during cancer evolution in these organs.

Finally, a significantly increased risk for distinct cancer forms among methylation carriers may question the potential for selective screening of these individuals. The prognosis of colon cancer, in particular, relies on early diagnosis, and the debate of selective colonoscopy-screening is ongoing.

The Women Health Initiative (WHI) is a unique study with a unique biobank, including normal tissue DNA from > 160.000 American women followed over 3 decades for incidental diseases, including cancers, with detailed information including histopathological confirmation of diagnosis. In collaboration with the WHI, we already have completed one successful study of mosaic gene methylation (*BRCA1*) as a predictor for subsequent incident cancers (main WHI investigator: Professor Rowan Chlebowski).

Laboratory analytical methods. The laboratory methods previously applied for promoter methylation studies have been suboptimal with respect to sensitivity as well as precision. None of the methods applied, including recent pyrosequencing (limited by short read lengths with limited sensitivity), could depict exact methylation pattern across individual cells (Figure 1), and methylation arrays, like the Illumina 450k, are restricted to cover only a limited number of CpGs across each promoter region.

To overcome these obstacles, we have developed a novel highly sensitive NGS-based assay, allowing detailing methylation patterns across individual cells (99). This assay has been customized, covering the individual genes needed for each analysis. This will allow high-

throughput analyses (96 samples on each of our two MiSeq instruments at any given time), yielding allele specific methylation calls at very high depth (25.000x).

Bioinformatics. Multiple bioinformatic tools for analysis of differential DNA methylation are available, however none of them account for potential existence of epialleles within a sample. To address their limitations we have developed a set of epiallele-aware bioinformatic tools, which are publicly available and can be easily employed by the scientific community. Among them, a method for discovery of low-frequency, low-magnitude aberrant methylation events in methylation array and next-generation sequencing data [https://github.com/BBCG/ramr, https://www.biorxiv.org/content/10.1101/2020.12.01.403501v1], and set of methods allowing to call hypermethylated epiallele frequencies at the level of genomic regions or individual cytosines, as well as to test the significance of the association between epiallele methylation status and single nucleotide polymorphisms (SNPs) [https://github.com/BBCG/epialleleR]. Recently, all details about our assay determining promoter methylation status of *MGMT* and *BRCA1* in the same run has been published and is openly available (100). Special focus during implementation of these methods was given not only to the functionality but also to their performance, resulting in excellent data processing speed, scalability and readiness to high-coverage whole-genome datasets.

Power analysis determining number of participants.

We will analyze *MGMT* promoter methylation in WBC samples collected at study inclusion from incident cases of colon cancer, glioblastomas and high-grade diffuse B-cell lymphomas and in matched controls drawn from the WHI biobank in a nested case-control design. With respect to **statistical power**, in preliminary analyses, measuring *MGMT* WBC methylation in umbilical cord blood from girls in the Norwegian Mother and child study (MoBa), we found WBC *MGMT1* methylation in 197/1260: 15.6% of newborn girls (95% CI: 13.7 -17.8%). From mothers (age 20-40), *MGMT* methylation was revealed among 100/575; 17.3.% (CI: 14.4-20.7%).

While some evidence indicates that *MGMT* methylation may occur more frequently in right- as compared to left-sided colon cancers (101), this is not firmly established. On the other hand, there is no evidence indicating *MGMT* methylation may occur at a higher frequency in left-sided colon cancer or rectal tumours as compared to right-sided / transversal cancers. Taking into account the biological differences between right- versus left-sided colon cancers, for this study, we will analyse *MGMT* methylation status in right-sided and left-sided colon cancers separately.

We will use our preliminary data for methylation / SNP rs16906252, obtained from young adults, as basis for estimates of statistical power. A modest correlation between the rs16906252 and promoter methylation has been reported previously (86, 87, 89, 92, 102). In our data set, among 575 individuals, 35 were methylated carrying the variant allele (6.1%), 49 carried the variant allele without *MGMT* methylation (8.5%), 65/575 were homozygous for the reference rs16906252 allele carrying *MGMT* methylation (11.3%), while 426/575 (74%) were homozygous for the reference allele and did not carry *MGMT* methylation.

The figures above allow testing potential effects of the SNP allele and *MGMT* methylation separately on colon cancer risk. For both tests, we assume a HR of at least 2.

Testing for the effect of *MGMT* methylation status among individuals not carrying the SNP variant. Removing all carriers of the SNP rs16906252 variant allele (among young women 84/575; 14.6%), less than 20% of participant in total will be omitted from the statistical analysis. In the group homozygous for the reference rs16906252 allele 65/491 carry *MGMT* methylation (13.2%). For a 1 : 2 matched nested case control design assuming a HR of 2 and

assuming a methylation frequency of 12% we will need 195 cancer patients (Table 1). Assuming a methylation frequency of 8%, this number increases to 310. Assuming up to 20% of all individuals to be either homo- or heterozygous for the variant allele, another 66 patients need to be added. Accounting for some samples being lost to analytical failure, starting with n = 400 patients with 800 controls in a 1 : 2 design will will provide an adequate sample size.

Testing for the effect of *MGMT* variant allele status among individuals not carrying *MGMT* promoter methylation. Out of 575 individuals, in total 100 were found methylated at the *MGMT* promoter (17.4%). Among the non-methylated individuals (82.6%; 475/575), 10.3%; (49/475) had the variant allele. Assuming the variant allele to be found among 8% (or more) of individuals, the calculations above will apply, confirming a total of n = 400 patients and 800 controls will be adequate for this test as well.

Taken together, we are able to test for the effect of *MGMT* methylation and the variant rs16906252 allele separately in nested case-control cohorts of 400 patients and 800 controls in a robust design. Thus, we aim testing for potential impact of *MGMT* methylation status and the rs16906252 variant allele on HR for right- and left-sided colon cancer (2 separate cohorts) with 400 patients and 800 matched controls for each comparison.

The fact that glioblastoma is a less common cancer form does not exclude constitutional *MGMT* methylation as a potential risk factor. As for glioblastomas, in the WHI study a total of n = 195 glioblastomas are so far recorded. Assuming a methylation frequency in the normal population of between 12 and 15% and a cancer OR of 2, a potential impact of *MGMT* promoter methylation may be tested in the total group with a power of 0.9 (Table 1), and the impact of *MGMT* methylation and rs16906252 variant allele status separately with a power between 0.8 and 0.9, assuming a 1 : 4 nested control design.

Considering diffuse large cell B-lymphomas, the WHI contains more than 500 cases. With the *MGMT* methylation frequencies recorded in the literature, we assume a HR and statistical power calculation resembling that performed for each of the two colon cancer groups. Thus, we will enroll n = 400 lymphoma cases with 800 matched controls.

Table 1. Overview of sample sizes required given $\alpha = 0.05$, $1\text{-}\beta = 0.8$ or 0.9, and OR in the range of 1.3 – 3.0, for different percentages of methylated healthy individuals (controls) in the population. The numbers listed are given as the number of cancer cases required, given a 1:2 mathced design (2 healthy controls per analyzed cancer case) or a 1:4 matched design

		Match 1 : 2			Match 1 : 4		
HR	Power (1-β	8 %	12 %	15 %	8 %	12 %	15 %
1.5	0.8	780	490	375	604	378	290
1.5	0.9	na	650	500	783	483	377
2.0	0.8	230	145	112	186	115	87
2.0	0.9	310	195	150	236	147	111
3.0	0.8	75	47	35	62	na	na
3.0	0.9	100	62	45	77	na	na

Summary of individuals to be enrolled. Based on the estimates above, for the colon cancer study we need to enroll a total of n = 800 participants with colon cancers and a total of n = 1.600 controls. For the glioblastoma study, we will enroll all 195 participants developing glioblastomas with n = 780 controls, while for the lymphoma study 400 cases and 800 controls will be included. **This adds to a total number of 4.575 individuals.** However, like for the previous *BRCA1* study, we assume several control individuals may act as controls in different comparisons; thus, most likely this number of control individuals may be reduced. In our previous study on breast- and ovarian cancers (WHI AS543), we were able to reduce the total number of controls by about 23% by using some of the same controls for both comparisons. As

for this study, we like to keep separate controls for the two colon-studies. However, leaving one of these out, assuming a reduction of controls of 20% among the rest, we may limit the number of controls by 476; thus, the total number of samples should be around 4.100.

Statistical analysis of data.

The potential association between methylation and risk of cancer will be performed by Cox multivariate analysis as the primary analysis. In addition, for sensitivity analysis we will perform logistic regression analysis. In both analyses, potential confounding variables will be included as co-variates, similar to our WHI AS543 study now in revised review in JAMA Oncology. The co-variates included will include age, race, previous hormone usage, DNA extraction method and geographical location. Methylation positivity for each sample will be determined based on presence fully methylated alleles (VEF), and the laboratory analysis conducted blinded to case control identity. Optimization of cutoff values will be performed using R package OptimalCutpoints (v1.1-4).(100).

Ethical Considerations.

The Women health Initiative (WHI) study has enrolled a cohort of 160.000 women to be followed for health issues over time. At enrollment, all participants provided blood samples and gave permission for analysis of different bio-parameters, including genomic analyses. Further, the patients were informed and consented to the principle no information from the laboratory data should be communicated at an individual level.

Apart from the Ethical Approvals of the WHI, this project has been granted approval by our regional Ethical Committee. Notably, the Norwegian investigators will have no access to individual ID. All samples (controls as well as samples obtained from women later developing cancers) are identified by study code only. All samples will be analyzed blinded; at the time of analyses, our laboratory staff will have no information identifying who are patients versus controls. The results for the coded samples will be transferred to the WHI database and handled in accordance with the WHI approvals and US regulations and laws. Thus, the logistics for data handling will mirror the handling previously approved for the AS543 study, and the ethical issues related to this project are fully similar to the questions addressed in respect to that previous project.

1. Ziegler RG, Hoover RN, Pike MC, Hildesheim A, Nomura AMY, West DW, et al. MIGRATION PATTERNS AND BREAST-CANCER RISK IN ASIAN-AMERICAN WOMEN. Jnci-Journal of the National Cancer Institute. 1993;85(22):1819-27.
2. Stanford JL, Herrinton LJ, Schwartz SM, Weiss NS. BREAST-CANCER INCIDENCE IN ASIAN MIGRANTS TO THE UNITED-STATES AND THEIR DESCENDANTS. Epidemiology. 1995;6(2):181-3.
3. Shuldiner J, Liu Y, Lofters A. Incidence of breast and colorectal cancer among immigrants in Ontario, Canada: a retrospective cohort study from 2004-2014. Bmc Cancer. 2018;18.
4. Hemminki K, Li X. Cancer risks in Nordic immigrants and their offspring in Sweden. European Journal of Cancer. 2002;38(18):2428-34.
5. Kucab JE, Zou XQ, Morganella S, Joel M, Nanda AS, Nagy E, et al. A Compendium of Mutational Signatures of Environmental Agents. Cell. 2019;177(4):821-+.
6. Haberle V, Stark A. Eukaryotic core promoters and the functional basis of transcription initiation. Nature Reviews Molecular Cell Biology. 2018;19(10):621-37.

7. Newell-Price J, Clark AJL, King P. DNA methylation and silencing of gene expression. Trends in Endocrinology and Metabolism. 2000;11(4):142-8.
8. Fahrner JA, Eguchi S, Herman JG, Baylin SB. Dependence of histone modifications and gene expression on DNA hypermethylation in cancer. Cancer Research. 2002;62(24):7213-8.
9. Fraga MF, Ballestar E, Paz MF, Ropero S, Setien F, Ballestart ML, et al. Epigenetic differences arise during the lifetime of monozygotic twins. Proceedings of the National Academy of Sciences of the United States of America. 2005;102(30):10604-9.
10. Ollikainen M, Smith KR, Joo EJH, Ng HK, Andronikos R, Novakovic B, et al. DNA methylation analysis of multiple tissues from newborn twins reveals both genetic and intrauterine components to variation in the human neonatal epigenome. Human Molecular Genetics. 2010;19(21):4176-88.
11. Ligtenberg MJL, Kuiper RP, Chan TL, Goossens M, Hebeda KM, Voorendt M, et al. Heritable somatic methylation and inactivation of MSH2 in families with Lynch syndrome due to deletion of the 3 ' exons of TACSTD1. Nature Genetics. 2009;41(1):112-7.
12. Galetzka D, Hansmann T, El Hajj N, Weis E, Irmscher B, Ludwig M, et al. Monozygotic twins discordant for constitutive BRCA1 promoter methylation, childhood cancer and secondary cancer. Epigenetics. 2012;7(1):47-54.
13. Ferrari L, Carugno M, Bollati V. Particulate matter exposure shapes DNA methylation through the lifespan. Clinical Epigenetics. 2019;11(1).
14. Rider CF, Carlsten C. Air pollution and DNA methylation: effects of exposure in humans. Clinical Epigenetics. 2019;11(1).
15. Hitchins MP. Constitutional epimutation as a mechanism for cancer causality and heritability? Nature Rev Cancer. 2015;15(10):181-94.
16. Sloane MA, Ward RL, Hesson LB. Defining the criteria for identifying constitutional epimutations. Clinical Epigenetics. 2016;8.
17. Hitchins MP. The role of epigenetics in Lynch syndrome. Familial Cancer. 2013;12(2):189-205.
18. Hesson LB, Hitchins MP, Ward RL. Epimutations and cancer predisposition: importance and mechanisms. Current Opinion in Genetics & Development. 2010;20(3):290-8.
19. Mazor T, Pankov A, Johnson BE, Hong CB, Hamilton EG, Bell RJA, et al. DNA Methylation and Somatic Mutations Converge on the Cell Cycle and Define Similar Evolutionary Histories in Brain Tumors. Cancer Cell. 2015;28(3):307-17.
20. Lønning PE, Eikesdal HP, Loes IM, Knappskog S. Constitutional Mosaic Epimutations - a hidden cause of cancer? Cell Stress. 2019;3(4):118-35.
21. Larsen IK, Bray F. Trends in colorectal cancer incidence in Norway 1962-2006: an interpretation of the temporal patterns by anatomic subsite. International Journal of Cancer. 2010;126(3):721-32.
22. Swerdlow AJ, DeStavola BL, Swanwick MA, Maconochie NES. Risks of breast and testicular cancers in young adult twins in England and Wales: Evidence on prenatal and genetic aetiology. Lancet. 1997;350(9093):1723-8.
23. Hitchins MP, Rapkins RW, Kwok CT, Srivastava S, Wong JJL, Khachigian LM, et al. Dominantly Inherited Constitutional Epigenetic Silencing of MLH1 in a Cancer-Affected Family Is Linked to a Single Nucleotide Variant within the 5 ' UTR. Cancer Cell. 2011;20(2):200-13.
24. Morak M, Koehler U, Schackert HK, Steinke V, Royer-Pokora B, Schulmann K, et al. Biallelic MLH1 SNP cDNA expression or constitutional promoter methylation can hide genomic rearrangements causing Lynch syndrome. Journal of Medical Genetics. 2011;48(8):513-9.
25. Gylling A, Ridanpaa M, Vierimaa O, Aittomaki K, Avela K, Kaariainen H, et al. Large genomic rearrangements and germline epimutations in Lynch syndrome. International Journal of Cancer. 2009;124(10):2333-40.
26. Evans DGR, van Veen EM, Byers HJ, Wallace AJ, Ellingford JM, Beaman G, et al. A Dominantly Inherited 5' UTR Variant Causing Methylation-Associated Silencing of BRCA1 as a Cause of Breast and Ovarian Cancer. American Journal of Human Genetics. 2018;103(2):213-20.

27. Lønning PE, Berge EO, Bjornslett M, Minsaas L, Chrisanthar R, Hoberg-Vetti H, et al. White Blood Cell BRCA1 Promoter Methylation Status and Ovarian Cancer Risk. Annals of Internal Medicine. 2018;168(5):326-+.
28. Liggett TE, Melnikov AA, Marks JR, Levenson VV. Methylation patterns in cell-free plasma DNA reflect removal of the primary tumor and drug treatment of breast cancer patients. International Journal of Cancer. 2011;128(2):492-9.
29. Rack B, Schindlbeck C, Andergassen U, Lorenz R, Zwingers T, Schneeweiss A, et al. Prognostic relevance of circulating tumor cells in the peripheral blood of primary breast cancer patients. Ca Res. 2010;70(Suppl)(24):S6-5, 93s.
30. Diehl F, Schmidt K, Choti MA, Romans K, Goodman S, Li M, et al. Circulating mutant DNA to assess tumor dynamics. Nature Medicine. 2008;14(9):985-90.
31. Anderson G, Cummings S, Freedman LS, Furberg C, Henderson M, Johnson SR, et al. Design of the Women's Health Initiative Clinical Trial and Observational Study. Controlled Clinical Trials. 1998;19(1):61-109.
32. Youssoufian H, Pyeritz RE. Mechanisms and consequences of somatic mosaicism in humans. Nature Reviews Genetics. 2002;3(10):748-58.
33. Zhang JH, Walsh MF, Wu G, Edmonson MN, Gruber TA, Easton J, et al. Germline Mutations in Predisposition Genes in Pediatric Cancer. New England Journal of Medicine. 2015;373(24):2336-46.
34. Evans GR, Ramsden RT, Shenton A, Gokhale C, Bowers NL, Huson SM, et al. Mosaicism in neurofibromatosis type 2: an update of risk based on uni/bilaterality of vestibular schwannoma at presentation and sensitive mutation analysis including multiple ligation-dependent probe amplification. Journal of Medical Genetics. 2007;44(7):424-8.
35. Friedman E, Efrat N, Soussan-Gutman L, Dvir A, Kaplan Y, Ekstein T, et al. Low-level constitutional mosaicism of a de novoBRCA1 gene mutation. British Journal of Cancer. 2015;112(4):765-8.
36. Delon I TA, Molenda A, Drummond J, Oakhill K, Girling A, Liu H, Whittaker J, Treacy R, Tischkowitz M. A germline mosaic BRCA1 exon deletion in a woman with bilateral basal-like breast cancer. Clin Genet 2012
37. Ainsworth PJ, Chakraborty PK, Weksberg R. Example of somatic mosaicism in a series of de novo neurofibromatosis type 1 cases due to a maternally derived deletion. Human Mutation. 1997;9(5):452-7.
38. Sippel KC, Fraioli RE, Smith GD, Schalkoff ME, Sutherland J, Gallie BL, et al. Frequency of somatic and germ-line mosaicism in retinoblastoma: Implications for genetic counseling. American Journal of Human Genetics. 1998;62(3):610-9.
39. LoTenFoe JR, Kwee ML, Rooimans MA, Oostra AB, Veerman AJP, vanWeel M, et al. Somatic mosaicism in Fanconi anemia: Molecular basis and clinical significance. European Journal of Human Genetics. 1997;5(3):137-48.
40. Marmol I, Sanchez-De-Diego C, Dieste AP, Cerrada E, Yoldi MJR. Colorectal Carcinoma: A General Overview and Future Perspectives in Colorectal Cancer. International Journal of Molecular Sciences. 2017;18(1).
41. Lynch HT, Snyder CL, Shaw TG, Heinen CD, Hitchins MP. Milestones of Lynch syndrome: 1895-2015. Nature Reviews Cancer. 2015;15(3):181-94.
42. Bucksch K, Zachariae S, Aretz S, Buttner R, Holinski-Feder E, Holzapfel S, et al. Cancer risks in Lynch syndrome, Lynch-like syndrome, and familial colorectal cancer type X: a prospective cohort study. Bmc Cancer. 2020;20(1).
43. Menahem B, Alves A, Regimbeau JM, Sabbagh C. Lynch Syndrome: Current management In 2019. Journal of Visceral Surgery. 2019;156(6):507-14.
44. Roberts ME, Jackson SA, Susswein LR, Zeinomar N, Ma XR, Marshall ML, et al. MSH6 and PMS2 germ-line pathogenic variants implicated in Lynch syndrome are associated with breast cancer. Genetics in Medicine. 2018;20(10):1167-74.
45. Lynch HT, de la Chapelle A. Genomic medicine - Hereditary colorectal cancer. New England Journal of Medicine. 2003;348(10):919-32.

46. Umar A, Boland CR, Terdiman JP, Syngal S, de la Chapelle A, Ruschoff J, et al. Revised Bethesda Guidelines for hereditary nonpolyposis colorectal cancer (Lynch syndrome) and microsatellite instability. Journal of the National Cancer Institute. 2004;96(4):261-8.
47. Sugai T, Yoshida M, Eizuka M, Uesugii N, Habano W, Otsuka K, et al. Analysis of the DNA methylation level of cancer-related genes in colorectal cancer and the surrounding normal mucosa. Clinical Epigenetics. 2017;9.
48. Li X, Yao XP, Wang YB, Hu FL, Wang F, Jiang LY, et al. MLH1 Promoter Methylation Frequency in Colorectal Cancer Patients and Related Clinicopathological and Molecular Features. Plos One. 2013;8(3).
49. Herman JG, Umar A, Polyak K, Graff JR, Ahuja N, Issa JPJ, et al. Incidence and functional consequences of hMLH1 promoter hypermethylation in colorectal carcinoma. Proceedings of the National Academy of Sciences of the United States of America. 1998;95(12):6870-5.
50. Cunningham JM, Christensen ER, Tester DJ, Kim CY, Roche PC, Burgart LJ, et al. Hypermethylation of the hMLH1 promoter in colon cancer with microsatellite instability. Cancer Research. 1998;58(15):3455-60.
51. Miyakura Y, Sugano K, Akasu T, Yoshida T, Maekawa M, Saitoh S, et al. Extensive but Hemiallelic Methylation of the hMLH1 Promoter Region in Early-Onset Sporadic Colon Cancers With Microsatellite Instability. Clinical Gastroenterology and Hepatology. 2004;2(2):147-56.
52. Boland CR, Thibodeau SN, Hamilton SR, Sidransky D, Eshleman JR, Burt RW, et al. A National Cancer Institute Workshop on Microsatellite Instability for Cancer Detection and Familial Predisposition: Development of International Criteria for the Determination of Microsatellite Instability in Colorectal Cancer. Cancer Research. 1998;58(22):5248-57.
53. Pasanen A, Loukovaara M, Butzow R. Clinicopathological significance of deficient DNA mismatch repair and MLH1 promoter methylation in endometrioid endometrial carcinoma. Modern Pathology. 2020.
54. Shikama A, Minaguchi T, Matsumoto K, Akiyama-Abe A, Nakamura Y, Michikami H, et al. Clinicopathologic implications of DNA mismatch repair status in endometrial carcinomas. Gynecologic Oncology. 2016;140(2):226-33.
55. Gazzoli I, Loda M, Garber J, Syngal S, Kolodner RD. A hereditary nonpolyposis colorectal carcinoma case associated with hypermethylation of the MLH1 gene in normal tissue and loss of heterozygosity of the unmethylated allele in the resulting microsatellite instability-high tumor. Cancer Research. 2002;62(14):3925-8.
56. Auclair J, Vaissiere T, Desseigne F, Lasset C, Bonadona V, Giraud S, et al. Intensity-Dependent Constitutional MLH1 Promoter Methylation Leads to Early Onset of Colorectal Cancer by Affecting Both Alleles. Genes Chromosomes & Cancer. 2011;50(3):178-85.
57. Hitchins M, Williams R, Cheong K, Halani N, Lin VA, Packham D, et al. MLH1 germline epimutations as a factor in hereditary nonpolyposis colorectal cancer. Gastroenterology. 2005;129(5):1392-9.
58. Suter CM, Martin DIK, Ward RL. Germline epimutation of MLH1 in individuals with multiple cancers. Nature Genetics. 2004;36(5):497-501.
59. Hitchins MP, Wong JJL, Suthers G, Suter CM, Martin DIK, Hawkins NJ, et al. Brief report: Inheritance of a cancer-associated MLH1 germ-line epimutation. New England Journal of Medicine. 2007;356(7):697-705.
60. Goel A, Nguyen T-P, Leung H-CE, Nagasaka T, Rhees J, Hotchkiss E, et al. De novo constitutional MLH1 epimutations confer early-onset colorectal cancer in two new sporadic Lynch syndrome cases, with derivation of the epimutation on the paternal allele in one. International Journal of Cancer. 2011;128(4):869-78.
61. Morak M, Schackert HK, Rahner N, Betz B, Ebert M, Walldorf C, et al. Further evidence for heritability of an epimutation in one of 12 cases with MLH1 promoter methylation in blood cells clinically displaying HNPCC. Eur J Hum Genet. 2008;16(7):804-11.
62. Morak M, Ibisler A, Keller G, Jessen E, Laner A, Gonzales-Fassrainer D, et al. Comprehensive analysis of the MLH1 promoter region in 480 patients with colorectal cancer and 1150 controls

reveals new variants including one with a heritable constitutional MLH1 epimutation. Journal of Medical Genetics. 2018;55(4):240-8.
63. Ward RL, Dobbins T, Lindor NLM, Rapkins RW, Hitchins MP. Identification of constitutional MLH1 epimutations and promoter variants in colorectal cancer patients from the Colon Cancer Family Registry. Genetics in Medicine. 2013;15(1):25-35.
64. Pinto D, Pinto C, Guerra J, Pinheiro M, Santos R, Vedeld HM, et al. Contribution of MLH1 constitutional methylation for Lynch syndrome diagnosis in patients with tumor MLH1 downregulation. Cancer Medicine. 2018;7(2):433-44.
65. Crepin M, Dieu MC, Lejeune S, Escande F, Boidin D, Porchet N, et al. Evidence of constitutional MLH1 epimutation associated to transgenerational inheritance of cancer susceptibility. Hum Mutat. 2012;33:180-8.
66. Niessen RC, Hofstra RMW, Westers H, Ligtenberg MJL, Kooi K, Jager POJ, et al. Germline Hypermethylation of MLH1 and EPCAM Deletions Are a Frequent Cause of Lynch Syndrome. Genes Chromosomes & Cancer. 2009;48(8):737-44.
67. Chan TL, Yuen ST, Kong CK, Chan YW, Chan ASY, Ng WF, et al. Heritable germline epimutation of MSH2 in a family with hereditary nonpolyposis colorectal cancer. Nature Genetics. 2006;38(10):1178-83.
68. Toyota M, Ahuja N, Ohe-Toyota M, Herman JG, Baylin SB, Issa JPJ. CpG island methylator phenotype in colorectal cancer. Proceedings of the National Academy of Sciences of the United States of America. 1999;96(15):8681-6.
69. Issa JP. CpG island methylator phenotype in cancer. Nat Rev Cancer. 2004;4(12):988-93.
70. Markowitz SD, Bertagnolli MM. Molecular Origins of Cancer: Molecular Basis of Colorectal Cancer. New England Journal of Medicine. 2009;361(25):2449-60.
71. Fu D, Calvo JA, Samson LD. Balancing repair and tolerance of DNA damage caused by alkylating agents. Nature Reviews Cancer. 2012;12(2):104-20.
72. Hegi ME, Diserens A, Gorlia T, Hamou M, de Tribolet N, Weller M, et al. MGMT gene silencing and benefit from temozolomide in glioblastoma. New England Journal of Medicine. 2005;352(10):997-1003.
73. Esteller M, Hamilton SR, Burger PC, Baylin SB, Herman JG. Inactivation of the DNA repair gene O6-methylguanine-DNA methyltransferase by promoter hypermethylation is a common event in primary human neoplasia. Cancer Res. 1999;59(4):793-7.
74. Sugai T, Habano W, Jiao YF, Tsukahara M, Takeda Y, Otsuka K, et al. Analysis of molecular alterations in left- and right-sided colorectal carcinomas reveals distinct pathways of carcinogenesis - Proposal for new molecular profile of colorectal carcinomas. Journal of Molecular Diagnostics. 2006;8(2):193-201.
75. Chirieac LR, Shen LL, Catalano PJ, Issa JP, Hamilton SR. Phenotype of microsatellite-stable colorectal carcinomas with CpG island methylation. American Journal of Surgical Pathology. 2005;29(4):429-36.
76. Neuville A, Nicolet C, Meyer N, Schneider A, Legrain M, Brigand C, et al. Histologic characteristics of non microsatellite-instable A colon adenomas correlate with distinct molecular patterns. Human Pathology. 2011;42(2):244-53.
77. Nagy E, Gajjar KB, Patel, II, Taylor S, Martin-Hirsch PL, Stringfellow HF, et al. MGMT promoter hypermethylation and K-RAS, PTEN and TP53 mutations in tamoxifen-exposed and non-exposed endometrial cancer cases. British Journal of Cancer. 2014;110(12):2874-80.
78. Cornel KMC, Wouters K, Van de Vijver KK, van der Wurff AAM, van Engeland M, Kruitwagen R, et al. Gene Promoter Methylation in Endometrial Carcinogenesis. Pathology & Oncology Research. 2019;25(2):659-67.
79. Al-Moghrabi N, Al-Showimi M, Al-Yousef N, Al-Shahrani B, Karakas B, Alghofaili L, et al. Methylation of BRCA1 and MGMT genes in white blood cells are transmitted from mothers to daughters. Clinical Epigenetics. 2018;10.

80. Deng GR, Chen AD, Hong J, Chae HS, Kim YS. Methylation of CpG in a small region of the hMLH1 promoter invariably correlates with the absence of gene expression. Cancer Research. 1999;59(9):2029-33.
81. Miyakura Y, Sugano K, Konishi F, Ichikawa A, Maekawa M, Shitoh K, et al. Extensive methylation of hMLH1 promoter region predominates in proximal colon cancer with microsatellite instability. Gastroenterology. 2001;121(6):1300-9.
82. Nakagawa H, Nuovo GJ, Zervos EE, Martin EW, Salovaara R, Aaltonen LA, et al. Age-related hypermethylation of the 5 ' region of MLH1 in normal colonic mucosa is associated with microsatellite-unstable colorectal cancer development. Cancer Research. 2001;61(19):6991-5.
83. Skvortsova K, Masle-Farquhar E, Luu PL, Song JZ, Qu WJ, Zotenko E, et al. DNA Hypermethylation Encroachment at CpG Island Borders in Cancer Is Predisposed by H3K4 Monomethylation Patterns. Cancer Cell. 2019;35(2):297-+.
84. Esteller M, Garcia-Foncillas J, Andion E, Goodman SN, Hidalgo OF, Vanaclocha V, et al. Inactivation of the DNA-repair gene MGMT and the clinical response of gliomas to alkylating agents. New England Journal of Medicine. 2000;343(19):1350-4.
85. Wiewrodt D, Nagel G, Dreimueller N, Hundsberger T, Perneczky A, Kaina B. MGMT in primary and recurrent human glioblastomas after radiation and chemotherapy and comparison with p53 status and clinical outcome. International Journal of Cancer. 2008;122(6):1391-9.
86. Rapkins RW, Wang F, Nguyen HN, Cloughesy TF, Lai A, Ha W, et al. The MGMT promoter SNP rs16906252 is a risk factor for MGMT methylation in glioblastoma and is predictive of response to temozolomide. Neuro-Oncology. 2015;17(12):1589-98.
87. Kristensen LS, Nielsen HM, Hager H, Hansen LL. Methylation of MGMT in malignant pleural mesothelioma occurs in a subset of patients and is associated with the T allele of the rs16906252 MGMT promoter SNP. Lung Cancer. 2011;71(2):130-6.
88. Kristensen LS, Treppendahl MB, Asmar F, Girkov MS, Nielsen HM, Kjeldsen TE, et al. Investigation of MGMT and DAPK1 methylation patterns in diffuse large B-cell lymphoma using allelic MSP-pyrosequencing. Scientific Reports. 2013;3.
89. Leng SG, Bernauer AM, Hong CB, Do KC, Yingling CM, Flores KG, et al. The A/G Allele of Rs16906252 Predicts for MGMT Methylation and Is Selectively Silenced in Premalignant Lesions from Smokers and in Lung Adenocarcinomas. Clinical Cancer Research. 2011;17(7):2014-23.
90. Kuroiwa-Trzmielina J, Wang F, Rapkins RW, Ward RL, Buchanan DD, Win AK, et al. SNP rs16906252C > T Is an Expression and Methylation Quantitative Trait Locus Associated with an Increased Risk of Developing MGMT-Methylated Colorectal Cancer. Clinical Cancer Research. 2016;22(24):6266-77.
91. Shen LL, Kondo Y, Rosner GL, Xiao LC, Hernandez NS, Vilaythong J, et al. MGMT promoter methylation and field defect in sporadic colorectal cancer. Journal of the National Cancer Institute. 2005;97(18):1330-8.
92. Candiloro ILM, Dobrovic A. Detection of MGMT Promoter Methylation in Normal Individuals Is Strongly Associated with the T Allele of the rs16906252 MGMT Promoter Single Nucleotide Polymorphism. Cancer Prevention Research. 2009;2(10):862-7.
93. Esteller M, Gaidano G, Goodman SN, Zagonel V, Capello D, Botto B, et al. Hypermethylation of the DNA repair gene O-6-methylguanine DNA methyltransferase and survival of patients with diffuse large B-cell lymphoma. Journal of the National Cancer Institute. 2002;94(1):26-32.
94. Uccella S, Cerutti R, Placidi C, Marchet S, Carnevali I, Bernasconi B, et al. MGMT methylation in diffuse large B-cell lymphoma: validation of quantitative methylation-specific PCR and comparison with MGMT protein expression. Journal of Clinical Pathology. 2009;62(8):715-23.
95. Shawky SA, El-Borai MH, Khaled HM, Guda I, Mohanad M, Abdellateif MS, et al. The prognostic impact of hypermethylation for a panel of tumor suppressor genes and cell of origin subtype on diffuse large B-cell lymphoma. Molecular Biology Reports. 2019;46(4):4063-76.
96. Rossi D, Capello D, Gloghini A, Franceschetti S, Paulli M, Bhatia K, et al. Aberrant promoter methylation of multiple genes throughout the clinico-pathologic spectrum of B-cell neoplasia. Haematologica. 2004;89(2):154-64.

97. Yates LR, Knappskog S, Wedge D, Farmery JHR, Gonzalez S, Martincorena I, et al. Genomic Evolution of Breast Cancer Metastasis and Relapse. Cancer Cell. 2017;32(2):169-+.
98. Yates LR, Gerstung M, Knappskog S, Desmedt C, Gundem G, Van Loo P, et al. Subclonal diversification of primary breast cancer revealed by multiregion sequencing. Nature Medicine. 2015;21(7):751-+.
99. Poduval DB, Ognedal E, Sichmanova Z, Valen E, Iversen GT, Minsaas L, et al. Assessment of tumor suppressor promoter methylation in healthy individuals. Clinical Epigenetics. 2020;12(1).
100. Nikolaienko O, Lønning PE, Knappskog S. epialleleR: an R/BioC package for sensitive allele-specific methylation analysis in NGS data. bioRxiv. 2022:2022.06.30.498213.
101. Menigatti M, Truninger K, Gebbers JO, Marbet U, Marra G, Schar P. Normal colorectal mucosa exhibits sex- and segment-specific susceptibility to DNA methylation at the hMLH1 and MGMT promoters. Oncogene. 2009;28(6):899-909.
102. Ogino S, Hazra A, Tranah GJ, Kirkner GJ, Kawasaki T, Nosho K, et al. MGMT germline polymorphism is associated with somatic MGMT promoter methylation and gene silencing in colorectal cancer. Carcinogenesis. 2007;28(9):1985-90.
