# Supplementary material for: MGMT epimutations and risk of incident cancer of the colon, glioblastoma multiforme, and diffuse large B cell lymphomas
Source: Clin Epigenetics. 2025 Feb 20;17:28. doi: 10.1186/s13148-025-01835-x (PMC11841191; doi:10.1186/s13148-025-01835-x)
Supplement: Supplementary file 2 — Additional file2: Analytical addendum [file 13148_2025_1835_MOESM2_ESM.pdf]

Protocol: Exploring *MGMT* constitutional methylation and the *MGMT* SNP rs16906252 as risk factors for cancer coli, glioblastoma and diffuse large B-cell lymphoma in the WHI AS721 study

August 17, 2022

Analytical addendum by January 30, 2024.

Summary: Preliminary, blind assessment of data obtained by *MGMT* methylation profiling in a complete set of samples (n=3925) revealed regional differences in methylation within the *MGMT* upstream area. This finding expands the biological understanding of the *MGMT* promoter region structure and function and may influence interpretation of results, similarly to what we have observed for *BRCA1* [1] where the upstream region next to *NBR2* did not influence TNBC/HGSOC HR contrasting the other parts of the *BRCA1* promoter analyzed. As this discovery was made prior to unblinding of case-control status, the analytical plan for comparison of cases and controls is adjusted as explained below.

The main studies assessing *MGMT* methylation status across different cancer forms providing a basis for this study are listed in the original protocol. For the cancers included in this study, between 20% and 40% of tumors have been reported methylated in the *MGMT* upstream area. As for the regions analyzed previously, there have been different approaches in the literature: most of the studies [2-6] have focused on a narrow region located within the *MGMT* gene body (exon 1 and part of intron 1, Figure 1), while some other studies [7, 8] assessed a wider region (including upstream, promoter areas). No direct evidence has been provided in favor of a differential methylation between the exon 1 and the rest of the assessed area.

In our current methylation profiling, a relatively large region is covered (GRCh38 chr10:129467118-129467477) that includes the downstream part of the *MGMT* promoter, entire exon 1 and an upstream part of intron 1. Examining the methylation data for the complete set of samples, we observed an apparent differences in methylation and its frequency between these three distinct structural elements of the *MGMT* gene (Figure 1).

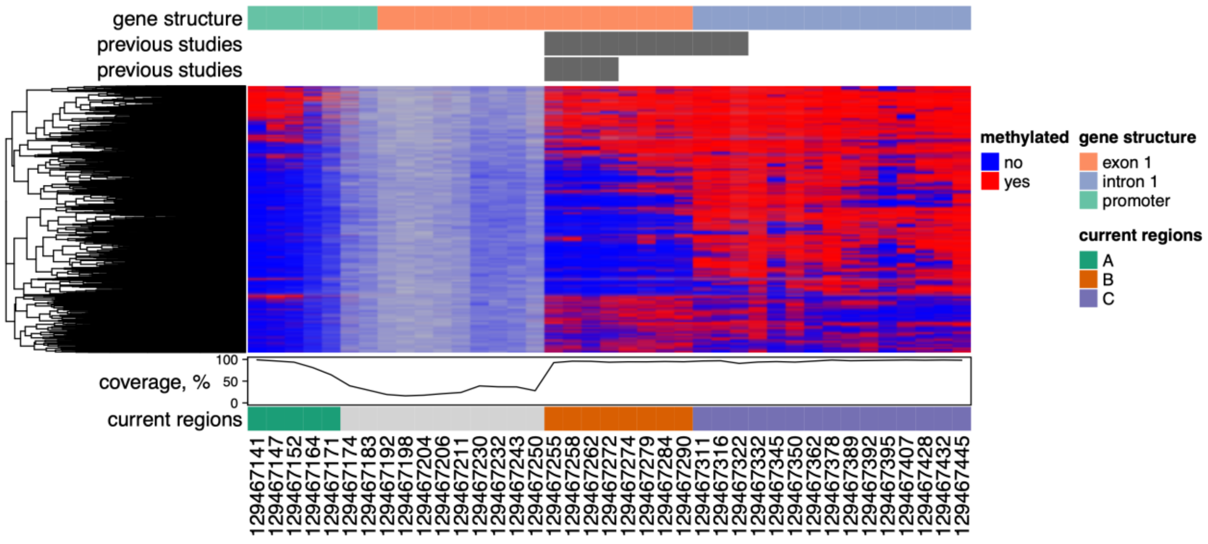

Figure 1. Top to bottom: structural elements of *MGMT* gene; regions covered by previous studies; heatmap of observed methylation patterns with average beta value of at least 0.25, clustered by pattern similarity (rows – patterns, columns – CpGs); sequencing coverage of individual CpGs normalized to total number of sequencing reads; regions defined in this addendum; genomic positions of CpGs covered by our assay (in ascending order).

Unsupervised hierarchical clustering of CpGs using methylation data allocates these structural elements to different clusters and thus confirms differences in their methylation properties (Figure 2).

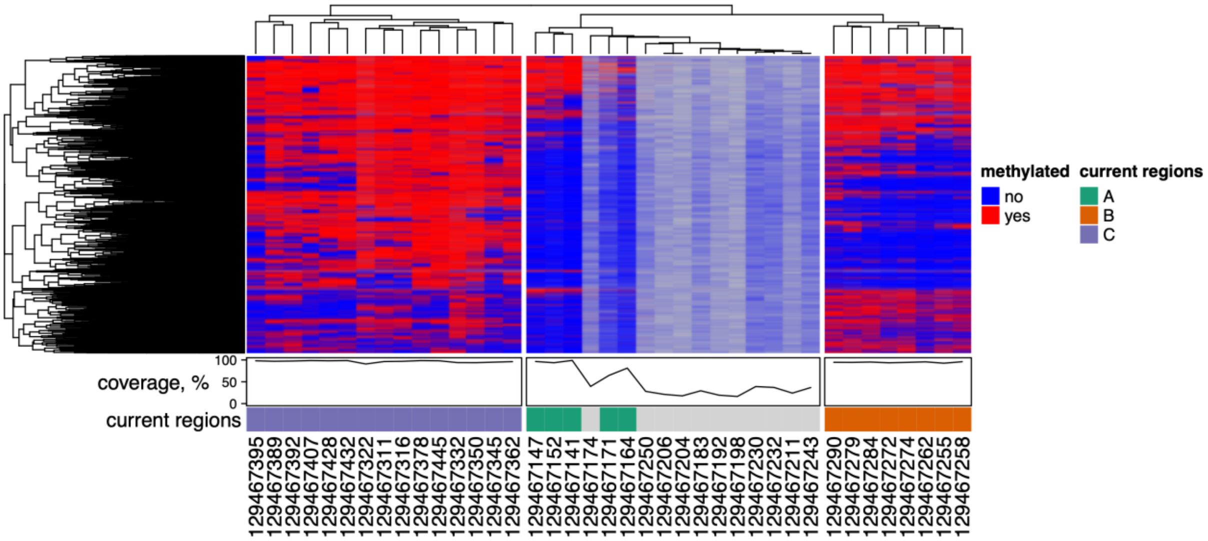

Figure 2. Same as Figure 1, but CpGs were clustered hierarchically and cut in three clusters. These clusters align well with *MGMT* structural elements, and three regions (A, B, and C) defined in Figure 1.

On the basis of these differences, we have defined three regions (covered by at least 50% of the sequencing reads; overlapping promoter, exon 1 and intron 1, and indicated as A, B and C, respectively; Figure 1). The amended analysis will therefore assess epimutation frequency and related cancer risk (HR) for these three regions separately.

For each of these regions, an epimutation will be defined as a "*methylation pattern with at least 75% of CpGs being informative, and more than a half of informative ones being methylated*", and epimutation-positive sample as a "*sample with the coverage of at least 10000x and epimutation frequency of at least 1/10000*". Primary analysis will focus of the region B overlapping *MGMT* exon 1, while secondary analysis will include regions A and C as well as combinations of regions A, B, and C. The analyses will assess cancer risk in all cases and controls, as well as in subgroups stratified by rs16906252 genotype and other variables of relevance.

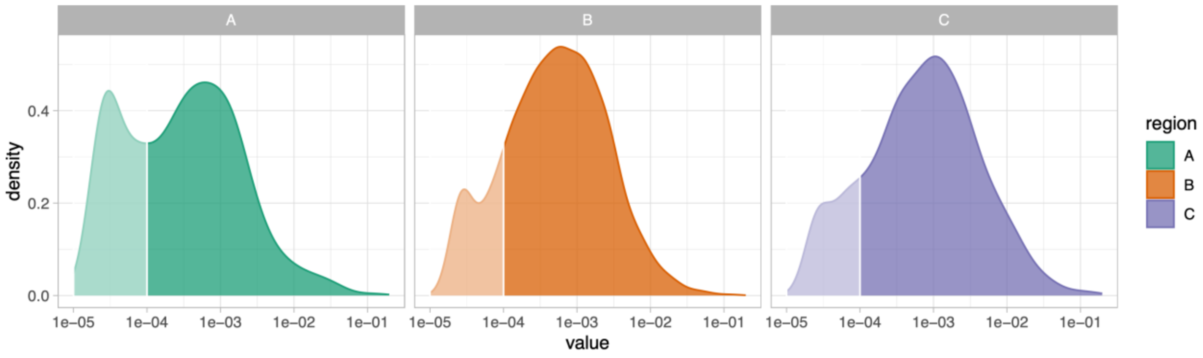

Figure 3. Density plots of observed per-sample frequencies of epimutations, split by regions. White vertical line represents the predefined cutoff for sample positivity (1e-4).

For the regions A, B and C combined, the analysis of the complete data set reveals a fraction of epimutation-positive samples of 8.1%, which aligns well with the estimates used in the original power analysis. The current assessment shows fraction of epimutation-positive samples of 9.4% across region A, 26.5% across region B, and 26.5% across region C. As for individuals being homozygous for the rs16906252 REF allele (87% of total number of individuals), fractions of epimutation-positive samples across areas A, B and C are 6.3%, 19.5% and 19.5%, respectively.

Varying fractions of epimutation-positive samples across regions A, B, and C will affect the statistical power of analyses. Given these fractions (above), the fraction of individuals homozygous for

rs16906252 REF allele of 0.87, and case/control ratios of 195:780 for glioblastoma (GB), 400:800 for diffuse large B-cell lymphoma (DLBCL), and 800:1600 for colorectal cancer (CRC), the statistical power is estimated as shown in Figure 4. Sufficient power (≥0.8) is retained for analysis of regions B and C for the OR≥1.5 in all cancers and in all groups of samples, as well as for the region A in colorectal cancer. Sufficient power for the analysis of region A in DLBCL is reached for OR≥1.5 in all samples and OR≥1.75 in rs16906252 REF/REF subgroup. Sufficient power for the analysis of region A in GB is reached for OR≥1.6 in all samples and OR≥1.85 in rs16906252 REF/REF subgroup.

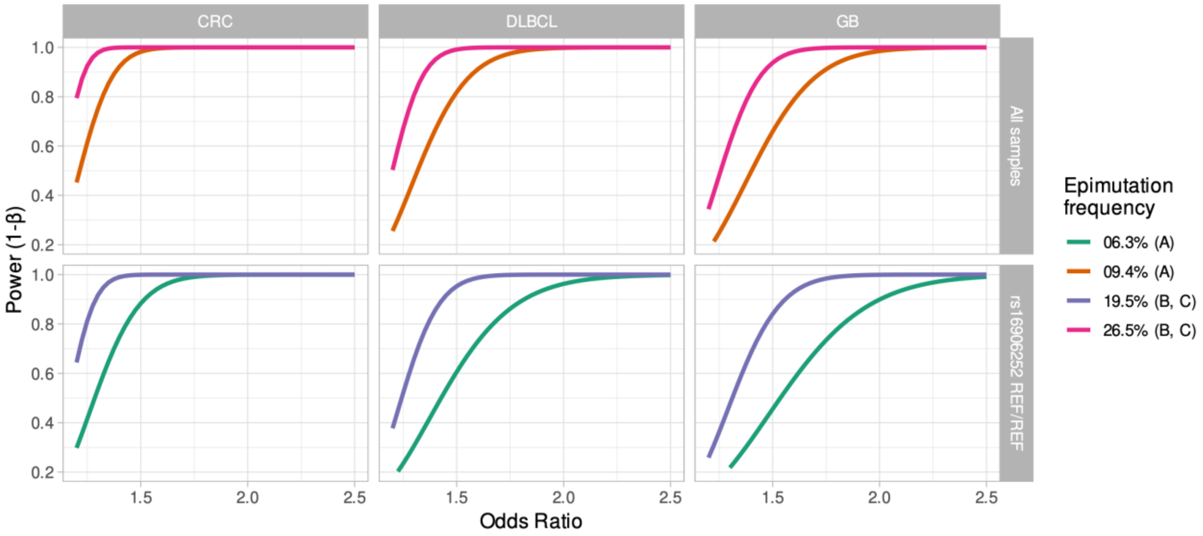

Figure 4. Statistical power (y-axis) for varying odds ratios (x axis) in three cancer types (columns), in subgroups of samples by rs16906252 genotype (all versus REF/REF; rows) for observed fractions of epimutation-positive samples (indicated by different colors).

1. Lønning PE, Nikolaienko O, Pan K, Kurian AW, Eikesdal HP, Pettinger M, Anderson GL, Prentice RL, Chlebowski RT, Knappskog S: **Constitutional BRCA1 Methylation and Risk of Incident Triple-Negative Breast Cancer and High-grade Serous Ovarian Cancer.** *Jama Oncology* 2022, **8:**1579-1587.
2. Esteller M, Hamilton SR, Burger PC, Baylin SB, Herman JG: **Inactivation of the DNA repair gene O6-methylguanine-DNA methyltransferase by promoter hypermethylation is a common event in primary human neoplasia.** *Cancer Res* 1999, **59:**793-797.
3. Estival A, Sanz C, Ramirez JL, Velarde JM, Domenech M, Carrato C, de las Penas R, Gil-Gil M, Sepulveda J, Armengol R, et al: **Pyrosequencing versus methylation-specific PCR for assessment of MGMT methylation in tumor and blood samples of glioblastoma patients.** *Scientific Reports* 2019, **9**.
4. Brawanski KR, Sprung S, Freyschlag CF, Hoeftberger R, Strobel T, Haybaeck J, Thome C, Manzl C, Birkl-Toeglhofer AM: **Influence of MMR, MGMT Promotor Methylation and Protein Expression on Overall and Progression-Free Survival in Primary Glioblastoma Patients Treated with Temozolomide.** *International Journal of Molecular Sciences* 2023, **24**.
5. Jensen GL, Pourfarrokh N, Volz M, Morales LL, Walker K, Hammonds KP, El-Ghamry M, Wong LC, Hodjat P, Castro E, Rao A, Jhavar SG: **Improved Pathologic response to chemoradiation in MGMT methylated locally advanced rectal cancer.** *Clinical and Translational Radiation Oncology* 2023, **42**.
6. Jimenez VG, Doval MB, Bellvert CG, Goliney VG, Asencio OS, Martin AG, Dominguez JI: **Quantitative analysis of MGMT promoter methylation status changes by pyrosequencing in recurrent glioblastoma.** *Neuropathology* 2023.

7. Watts GS, Pieper RO, Costello JF, Peng YM, Dalton WS, Futscher BW: **Methylation of discrete regions of the O-6-methylguanine DNA methyltransferase (MGMT) CpG island is associated with heterochromatinization of the MGMT transcription start site and silencing of the gene.** *Molecular and Cellular Biology* 1997, **17:**5612-5619.
8. Qian XLC, Brent TP: **Methylation hot spots in the 5' flanking region denote silencing of the O-6-methylguanine-DNA methyltransferase gene.** *Cancer Research* 1997, **57:**3672-3677.
