# Supplementary material for: MGMT epimutations and risk of incident cancer of the colon, glioblastoma multiforme, and diffuse large B cell lymphomas
Source: Clin Epigenetics. 2025 Feb 20;17:28. doi: 10.1186/s13148-025-01835-x (PMC11841191; doi:10.1186/s13148-025-01835-x)
Supplement: Supplementary file 3 — Additional file3: Sample selection protocol WHI [file 13148_2025_1835_MOESM3_ESM.docx]

July 25, 2023

**WHI Ancillary Study #721** Exploring MGMT constitutional methylation and the MGMT SNP rs16906252 as risk factors for cancer coli, glioblastomas and diffuse large B-cell lymphoma in the WHI study.

Study population: WHI Clinical Trial and Observational Study Participants (n=161,808)

Cancer outcomes and follow-up data as of 2/19/2023

Specimen requirements: Baseline DNA 1 ug @ 25 ng/ul

Case definition:

1. Glioblastoma: WHI outcome of brain cancer with histology code 9401/3, 9440/3, or 9442/3.
2. Diffuse large B-cell lymphoma (DLBCL): WHI outcome of non-Hodgins lymphoma or leukemia, with histology code 9680/3, 9684/3, 9678/3, 9679/3.
3. Colon: WHI colon cancer outcome with histology code 8140/3, 8210/3, 8261/3, 8263/3, or 8480/3. Right side, ICD 9 sites code 18.0, 18.2, or 18.3. Left side, ICD 9 site code 18.5, 18.6, or 18.7.

Exclusions

|  | Glioblastoma, n=221 | DLBCL, n=602 | Colon, n=1478 right side and n=637 left side, total n=2115 |
| --- | --- | --- | --- |
| Insufficient DNA from baseline blood draw | 5 | 16 | 71 |
| Missing smoking status at WHI baseline | 1 | 3 | 30 |
| History of brain cancer at WHI baseline | 1 | NA | NA |
| Leukemia, non-Hodgkin’s or Hodgkin’s lymphoma at baseline or during follow-up, prior to incident DLBCL | NA | 15 | NA |
| History of colorectal cancer, ulcerative colitis or Crohn's disease at baseline, or other colorectal cancer during follow-up, prior to incident colon cancer | NA | NA | 63 |
|  |  |  |  |
| Remaining eligible cases | n=214 | N=568 | N=2022 |

Note: There were nine participants with more than one of these cancers. They are included in the case count for each diagnosis. See details below.

Control sample:

Eligible controls were drawn from the CT+OS after excluding all cases of glioblastoma, DLBCL, or colon cancer.

Controls were further excluded from eligibility for the following reasons:

1. No follow-up
2. No DNA
3. Unknown smoking history.
4. Exclusions specific to Glioblastoma and DLBCL controls: History of brain, leukemia, Hodgkin’s lymphoma or non-Hodgkin’s lymphoma cancer reported at baseline or adjudicated during follow-up
5. Exclusions specific to Colon cancer controls: History of colorectal cancer, ulcerative colitis or Crohn's disease at baseline, or colorectal cancer during follow-up

**Number of eligible controls for glioblastoma and DLBCL: n=143,850**

**Number of eligible controls for colon cancer: n=139,478**

Case-control matching (Glioblastoma 1:4; DLBCL 1:2; right side colon 1:2; left side colon 1:2)

Matching criteria:

1. Age (± 3 years)
2. Smoking status at WHI baseline (Never, past, current), exact match
3. Race/ethnicity, exact match
4. DNA extraction method, exact match (if no extracted DNA, method= Qiagen/Five Prime)
5. Alive at time of index case diagnosis
6. Hormone therapy use categorized as never/ever estrogen alone/ever estrogen+progestin, where estrogen+progestin use takes precedence over estrogen alone use (HT_type coded as 0=never, 1=ever Ealone, 2=ever E+P). For cases, HT_type is defined during time interval prior to diagnosis date, which includes use reported at baseline. Controls are matched such that they had matching HT_type at any time prior to the index case diagnosis date.

Control selection was done in a time-forward manner, selecting controls for each case from the risk set at the time of the case’s event. The matching algorithm will be allowed to select the closest match based on a criteria to minimize an overall distance measure (Bergstralh EJ, Kosanke JL. Computerized matching of cases to controls. Technical Report #56, Department of Health Sciences Research, Mayo Clinic, Rochester MN. April 1995). Each matching factor will be given the same weight. Controls were first matched to glioblastoma cases. We then included these controls in the matching for DLBCL cases, in order to utilize as many controls as possible in a double manner, i.e. matched to both a glioblastoma and DLBCL case, however a case cannot be a control for another case. Controls for colon cancer cases were chosen specifically for those cases, with no overlap.

Matching results

1. Glioblastoma cases were matched first. N=214 cases with a goal of 4 matched control per case.
2. Never used HT prior to dx, N=73 cases

Total # controls = 292

1. Used HT prior to dx, N= 141 cases

Total # controls = 564

For glioblastoma, a total of 856 controls were matched to 214 cases.

Randomly selected 195 sets from the remaining 214 matched sets, prioritizing the inclusion of 2 case-control sets where glioblastoma case also had colon cancer or DLBCL.

Final selection for glioblastoma included 195 cases and 780 controls.

1. DLBCL cases were matched next; N=568 cases with a goal of 2 controls per case, including as much control overlap as possible with glioblastoma controls.
2. Never used HT prior to dx, N=171

Step 1. Using glioblastoma controls, matched 253 controls to 130 cases.

Step 2. Using remaining control pool, matched additional 7 controls to 7 cases above that only had one match in Step 1. Matched 82 controls to 41 cases that didn’t receive matched in Step 1.

Total of 171 cases and 684 cases in this step.

1. HT prior to dx, N=397

Step 1. Using glioblastoma controls, matched 490 controls to 259 cases.

Step 2. Using remaining control pool, matched additional 28 controls to 28 cases in Step 1 that had only one match in Step 1. Matched 274 controls to 137 cases with no glioblastoma control matches.

1. One case remained unmatched.

Total of 1134 controls matched to 567 cases.

Randomly selected 400 sets from the remaining 567 matched sets, prioritizing the inclusion of eight sets where DLBCL case also had glioblastoma or colon cancer

Final selection for DLBCL included 400 cases and 800 controls.

N=474 DLBCL controls were also selected as glioblastoma controls

1. Colon cancer cases were matched last; goal was 400 right side colon cancer cases and 400 left side colon cancer cases, with 2 controls individually matched to each, with no overlap in controls.

Because there were ample colon cases, we randomly selected n=405 right side cases and n=405 left side cases to do the matching, retaining 8 cases with colon cancer who also had diagnoses of glioblastoma or DLBCL.

The control pool did not include controls that were selected as glioblastoma and/or DLBCL controls.

1. Never used HT prior to dx, N=342 colon cases (total)

Matched 684 controls to n=342 cases.

1. Used HT prior to dx, N= 468 colon cases (total)

Matched 934 controls to n=467 cases.

Combining A and B above, a total of N=1618 controls were matched to N=809 cases (n=405 right side and n=404 left side). Finally, 400 cases and their matched controls were randomly selected from each group (right side or left side) for a total of 800 colon cases and 1600 controls. All colon cases that also had diagnoses of glioblastoma or DLBCL were retained.

Characteristics of Selected Cases

|  | Glioblastoma, N=195 | DLBCL, N=400 | Right side colon, N=400 | Left side colon, N=400 |
| --- | --- | --- | --- | --- |
| Age at blood draw, 5-year distribution,  N (%) |  |  |  |  |
| < 55 | 19 (9.7%) | 38 (9.5%) | 18 (4.5%) | 33 (8.3%) |
| 55 – 59 | 39 (20.0%) | 63 (15.8%) | 62 (15.5%) | 70 (17.5%) |
| 60 – 64 | 51 (26.2%) | 103 (25.8%) | 94 (23.5%) | 98 (24.5%) |
| 65 – 69 | 47 (24.1%) | 88 (22.0%) | 107 (26.8%) | 95 (23.8%) |
| 70 – 74 | 27 (13.8%) | 81 (20.2%) | 86 (21.5%) | 66 (16.5%) |
| ≥75 | 12 (6.2%) | 27 (6.8%) | 33 (8.3%) | 38 (9.5%) |
|  |  |  |  |  |
| Race/ethnicity, N (%) |  |  |  |  |
| White | 183 (93.9%) | 364 (91.0%) | 340 (85.0%) | 334 (83.5%) |
| Black | 4 (2.0%) | 8 (2.0%) | 34 (8.5%) | 37 (9.2%) |
| Hispanic | 4 (2.0%) | 13 (3.3%) | 10 (2.5%) | 10 (2.5%) |
| American Indian | 0 | 1 (0.2%) | 3 (0.8%) | 0 |
| Asian/PI | 2 (1.0%) | 6 (1.5%) | 7 (1.8%) | 11 (2.8%) |
| Unknown | 2 (1.0%) | 8 (2.0%) | 6 (1.5%) | 8 (2.0%) |
|  |  |  |  |  |
| Smoking, N (%) |  |  |  |  |
| Never | 92 (47.2%) | 214 (53.5%) | 186 (46.5%) | 201 (50.2%) |
| Past | 89 (45.6%) | 172 (43.0%) | 183 (45.8%) | 173 (43.2%) |
| Current | 14 (7.2%) | 14 (3.5%) | 31 (7.8%) | 26 (6.5%) |
|  |  |  |  |  |
| WHI Study Component |  |  |  |  |
| Observational study | 109 (55.9%) | 235 (58.8%) | 225 (56.2%) | 199 (49.8%) |
| Clinical Trial | 86 (44.1%) | 165 (41.2%) | 175 (43.8%) | 201 (50.2%) |
| Hormone therapy trial |  |  |  |  |
| Active (estrogen alone or estrogen+progestin) | 11 (12.8%) | 39 (23.6%) | 26 (14.8%) | 45 (22.4%) |
| Placebo | 16 (18.6%) | 23 (13.9%) | 43 (24.6%) | 36 (17.9%) |
| Not randomized to trial | 59 (68.6%) | 103 (62.4%) | 106 (60.6%) | 120 (59.7%) |
|  |  |  |  |  |
| Hormone therapy use prior to diagnosis date |  |  |  |  |
| Never used | 64 (32.8%) | 140 (35.0%) | 168 (42.0%) | 171 (42.8%) |
| Ever used estrogen alone | 58 (29.7%) | 122 (30.5%) | 121 (30.3%) | 120 (30.0%) |
| Ever used estrogen+progestin | 73 (37.4%) | 138 (34.5%) | 111 (27.8%) | 109 (27.2%) |
|  |  |  |  |  |
| DNA Extraction Method, n (%) |  |  |  |  |
| No extracted DNA | 143 (73.3%) | 141 (35.2%) | 254 (63.5%) | 264 (66.0%) |
| Extracted using Qiagen/Five Prime | 45 (23.1%) | 244 (61.0%) | 128 (32.0%) | 118 (29.5%) |
| Extracted using Phenol Chloroform | 6 (3.1%) | 12 (3.0%) | 13 (3.3%) | 13 (3.2%) |
| Extracted using Salt-precipitation | 1 (0.5%) | 3 (0.8%) | 5 (1.2%) | 5 (1.2%) |
| Extracted using Qiagen/Bioserve | 0 | 0 | 0 | 0 |

Nine cases had more than one type of cancer being studied. They were counted in each of the applicable columns above and below. There were 6 cases with both right side colon and DLBCL, 1 case with left side colon and DLBCL, 1 case with DLBCL and glioblastoma, and 1 case with right side colon and glioblastoma.

Tumor Characteristics of Selected Cases

| **Glioblastoma** | | | |
| --- | --- | --- | --- |
| Histology | Description | N | % |
| 9401/3 | ASTROCYTOMA ANAPLASTIC | 19 | 10.3 |
| 9440/3 | GLIOBLASTOMA NOS | 173 | 88.7 |
| 9442/3 | GLIOSARCOMA | 2 | 1.0 |
| Total |  | 195 |  |

| **Diffuse large B-cell lymphoma** | | | |
| --- | --- | --- | --- |
| Histology | Description | N | % |
| 9679/3 | MEDIASTINAL LARGE B-CELL LYMPHOMA | 1 | 0.3 |
| 9680/3 | LYMPHOMA, LARGE CELL DIFFUSE, NOS | 393 | 98.2 |
| 9684/3 | LYMPHOMA, IMMUNOBLASTIC, NOS | 6 | 1.5 |
| Total |  | 400 |  |

| **Right side colon** | | | |
| --- | --- | --- | --- |
| Histology | Description | N | % |
| 8140/3 | ADENOCARCINOMA NOS | 259 | 64.8 |
| 8210/3 | ADENOCARCINOMA IN ADENOMATOUS POLYP | 37 | 9.2 |
| 8261/3 | ADENOCARCINOMA IN VILLOUS ADENOMA | 9 | 2.2 |
| 8263/3 | ADENOCARCINOMA IN TUBULOVILLOUS ADENOMA | 42 | 10.5 |
| 8480/3 | MUCOUS ADENOCARCINOMA | 53 | 13.2 |
|  | **Right side colon (continued)** |  |  |
| ICD9 site | Description |  |  |
| 18.0 | Cecum | 186 | 46.5 |
| 18.2 | Ascending colon | 172 | 43.0 |
| 18.3 | Hepatic flexure of colon | 42 | 10.5 |
| Total |  | 400 |  |
| **Left side colon** | | | |
| Histology | Description | N | % |
| 8140/3 | ADENOCARCINOMA NOS | 267 | 66.8 |
| 8210/3 | ADENOCARCINOMA IN ADENOMATOUS POLYP | 50 | 12.5 |
| 8261/3 | ADENOCARCINOMA IN VILLOUS ADENOMA | 14 | 3.5 |
| 8263/3 | ADENOCARCINOMA IN TUBULOVILLOUS ADENOMA | 43 | 10.8 |
| 8480/3 | MUCOUS ADENOCARCINOMA | 26 | 6.5 |
|  |  |  |  |
| ICD9 site | Description |  |  |
| 18.5 | Splenic flexure of colon | 40 | 10.0 |
| 18.6 | Descending colon | 73 | 18.2 |
| 18.7 | Sigmoid colon | 287 | 71.8 |
| Total |  | 400 |  |
