# Supplementary material for: MGMT epimutations and risk of incident cancer of the colon, glioblastoma multiforme, and diffuse large B cell lymphomas
Source: Clin Epigenetics. 2025 Feb 20;17:28. doi: 10.1186/s13148-025-01835-x (PMC11841191; doi:10.1186/s13148-025-01835-x)
Supplement: Supplementary file 4 — Additional file4: Additional methods and results [file 13148_2025_1835_MOESM4_ESM.docx]

**Additional file 4**

**Supplementary Methods.**

**Supplementary Figure S1. The genomic structure of the *MGMT* promoter region.**

**Supplementary Table S1. GRCh38 genomic coordinates of the PCR amplicon and individual CpGs**

**Supplementary Results. Supplementary Figure S2. Average depth of coverage and methylation of *MGMT* promoter**

**Supplementary Figure S3. Observed versus expected methylation levels in control samples.**

**Supplementary Figure S4. Distribution of methylation beta values as compared to VEF values**

**Supplementary Figure S5. Histogram of VEF values for the assessed *MGMT* promoter regions**

**Supplementary Figure S6. Histogram of VEF values for GB cases and controls by region and rs16906252 genotype**

**Supplementary Figure S7. Histogram of VEF values for DLBCL cases and controls by region and rs16906252 genotype**

**Supplementary Figure S8. Histogram of VEF values for all CC cases and controls by region and rs16906252 genotype**

**Supplementary Figure S9. Histogram of VEF values for left-sided CC cases and controls by region and rs16906252 genotype**

**Supplementary Figure S10. Histogram of VEF values for right-sided CC cases and controls by region and rs16906252 genotype**

**Supplementary Figure S11. Risk for glioblastoma by region and rs16906252 genotype**

**Supplementary Figure S12. Risk for DLBCL by region and rs16906252 genotype**

**Supplementary Figure S13. Risk for left-sided colon cancer by region and rs16906252 genotype**

**Supplementary Figure S14. Risk for right-sided colon cancer by region and rs16906252 genotype**

**Supplementary Figure S15. Risk for colon cancer by region and rs16906252 genotype**

**Supplementary Table S2. Frequency of rs16906252 genotypes in cases and controls**

**Supplementary Table S3. Frequency of rs16906252 genotypes in methylation-positive and negative samples**

**Supplementary Figure S16. Risk for glioblastoma by promoter region and its methylation status**

**Supplementary Figure S17. Risk for DLBCL by promoter region and its methylation status**

**Supplementary Figure S18. Risk for left-sided colon cancer by promoter region and its methylation status**

**Supplementary Figure S19. Risk for right-sided colon cancer by promoter region and its methylation status**

**Supplementary Figure S20. Risk for colon cancer by promoter region and its methylation status**

**Supplementary Figure S21. Preferential methylation of ALT-allele of rs16906252**

**Supplementary References.**

This supplemental material has been provided by the authors to give readers additional information about their work.

**Supplementary Methods**

**Statistical power estimates**

A detailed description of estimates for statistical power is given in the attached study protocol **(Additional file 1)**. In brief, power estimates were based on previous results of *MGMT* methylation frequency among patients diagnosed with glioblastoma (GB), diffuse large B-cell lymphoma (DLBCL), or colon cancer (CC), and healthy controls. Assuming a hypermethylation frequency of about 12% among healthy individuals and a hazard ratio (HR) of 2, matching 200 GB on a 1:4 basis with 800 control samples in a nested design provides a power (1-β) of 0.9. For DLBCL, comparing n=400 patients on a 1:2 basis with 800 controls provides a 1-β of 0.9. For CC, comparing n=400 patients with left-sided and n=400 patients with right-sided carcinoma on a 1:2 basis with 1,600 controls provides a 1-β of 0.9. As such, knowing the availability of >200 GB cases, >600 DLBCL cases, and >2000 CC cases in the WHI biobank, we found these designs to provide adequate power to test the hypothesis.

**Selection of cases and case-controls matching**

Cases of GB, DLBCL, and CC were selected from the Women’s Health Initiative (WHI) Clinical Trial and Observational Study Participants (n=161,808) according to follow-up data as of 2/19/2023. All details on included sub-diagnoses and matching of controls are given in **Additional file 3**. In brief, a formal selection of n=195 GB, n=400 DLBCL, and n=800 CC cases was made, together with n=2,730 controls, out of which a fraction served as controls versus both GB and DLBCL (see below). Subsequent to selection, samples with too low DNA concentration were removed together with those matched controls that were selected on the basis of being matched to cases with too low DNA concentration. Thus, the final sample set consisted of 3,715 samples, out of which 178 were GB cases, 387 were DLBCL cases, 749 were CC cases (373 left-sided and 376 right-sided), and 2,410 were controls. 1 sample was from an individual with both a GB and a DLBCL diagnosis, 1 sample was from an individual with both a GB and a CC diagnosis, and 7 samples were from individuals with both a CC and a DLBCL diagnosis. Out of the 2,410 controls, 440 served as controls for both GB and DLBCL (**see Figure 1 in the main text**).

**WHI DNA Storage, Processing and QA**

Entry blood samples were obtained after at least 12 hours of fasting via a prespecified protocol standardized across all study sites. Blood samples were processed, buffy coat aliquoted and stored in freezers at −70°C within 2 hours of collection and shipped on dry ice to a central processing facility (Fisher Bioservices) where storage at −80°C was maintained.

To maintain consistency in handling, all specimen processing (DNA extraction and specimen aliquoting) occurs in the central WHI lab (Fred Hutch Specimen Processing Lab). Since 2008, the WHI DNA extraction procedure is the manual 5-Prime procedure. Prior to 2008, WHI used 3 other methods of extraction: Bioserve, salt precipitation, and phenol/chloroform. 85% of the samples in this study were extracted using the 5-Prime method. DNA was quantified by fluorescence (PicoGreen). For the samples extracted early in the study (prior to 2007), DNA concentration was measured spectrophotometrically with the 260/280 OD ratio.

To ensure that sufficient sample arrives in the testing lab, WHI does not generally distribute aliquots that are < 1 ug and does not generally dilute DNA to a concentration less than 50 ng/ul. Some DNA samples are less than 50 ng/ul upon extraction. They were provided as-is if they met the testing lab’s minimum requirements. If a sample was quantitated using the OD ratio, twice as much will be provided to ensure there is enough DNA.

All studies requesting DNA samples are required to include blind duplicates as quality control samples: 5% of the total number of participant samples (2.5% blind duplicate pairs). The study investigator is required to report to the WHI all duplicates identified. From this list, the WHI will confirm the correct/incorrect identification of the blind duplicates and any unexpected duplicates.

Human HCT116 DKO Non-Methylated and Methylated DNA control samples (Zymo Research, cat.no. D5014-1 and D5014-2 respectively) and their mixes with varying ratios were used to test assay sensitivity.

**Region of the *MGMT* promoter selected for analyses and assay design**

Previous studies have used different approaches to quantify *MGMT* promoter methylation: most of the studies (1-5) have focused on a narrow region located within the *MGMT* gene body (exon 1 and part of intron 1, Supplementary Figure S1), while some other studies (6-7) assessed a wider region (including upstream promoter areas). No direct evidence has been provided in favor of one versus the other assessed regions.

In the present study, a relatively large region is covered (GRCh38 chr10:129467118-129467477), that includes the downstream part of the *MGMT* promoter, the entire exon 1 and an upstream part of intron 1 (Supplementary Figure S1). These three *MGMT* subregions were labeled A, B and C, respectively (Supplementary Figure S1) and were assessed both separately and merged in statistical analyses, with region B as the primary analysis, as per study protocol with amendment (Additional files 1 and 2). A detailed rationale for this is provided in the protocol amendment (Additional file 2).

**Supplementary Figure S1. The genomic structure of the *MGMT* promoter region.**


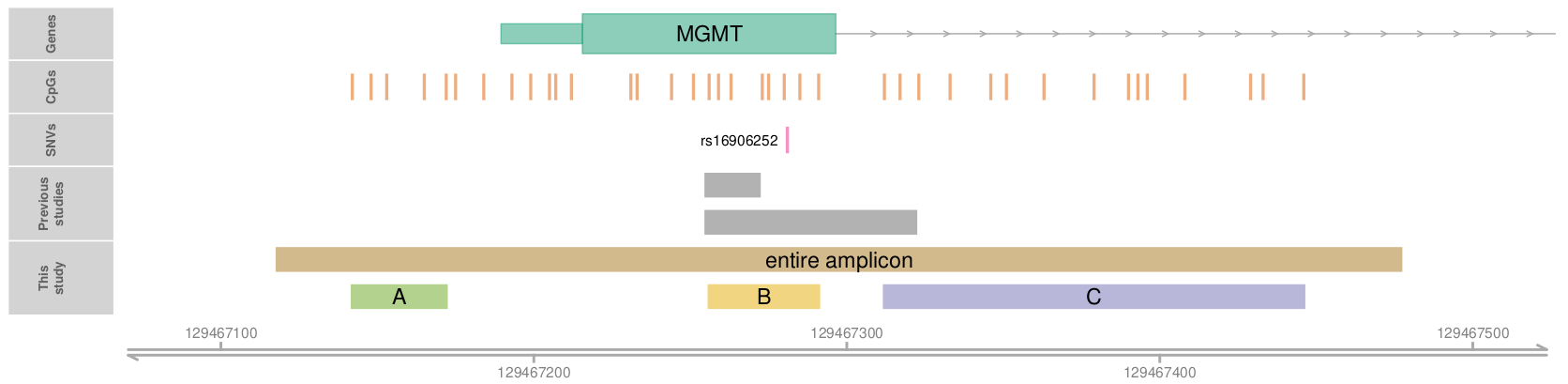
The first *MGMT* exon is depicted by green rectangles, where the thin left part represents 5’UTR. The covered CpGs are indicated by orange vertical lines. The single-nucleotide variant rs16906252 is indicated by a pink vertical line. Regions commonly analyses in other studies are indicated by gray rectangles. The amplicon and the three analyzed regions in the present study are indicated at the bottom by brown, light green, yellow and purple rectangles.

The assay applied in the present study covered 39 CpGs. The genomic coordinates for the complete amplicon and individual CpGs are given in Supplementary Table S1.

**Supplementary Table S1. GRCh38 genomic coordinates of the PCR amplicon and individual CpGs**

| **Type** | **Region** | **Coordinates** |
| --- | --- | --- |
| amplicon |  | chr10:129467118-129467477 |
| CpG | A | chr10:129467142 |
| CpG | A | chr10:129467148 |
| CpG | A | chr10:129467153 |
| CpG | A | chr10:129467165 |
| CpG | A | chr10:129467172 |
| CpG |  | chr10:129467175 |
| CpG |  | chr10:129467184 |
| CpG |  | chr10:129467193 |
| CpG |  | chr10:129467199 |
| CpG |  | chr10:129467205 |
| CpG |  | chr10:129467207 |
| CpG |  | chr10:129467212 |
| CpG |  | chr10:129467231 |
| CpG |  | chr10:129467233 |
| CpG |  | chr10:129467244 |
| CpG |  | chr10:129467251 |
| CpG | B | chr10:129467256 |
| CpG | B | chr10:129467259 |
| CpG | B | chr10:129467263 |
| CpG | B | chr10:129467273 |
| CpG | B | chr10:129467275 |
| CpG | B | chr10:129467280 |
| CpG | B | chr10:129467285 |
| CpG | B | chr10:129467291 |
| CpG | C | chr10:129467312 |
| CpG | C | chr10:129467317 |
| CpG | C | chr10:129467323 |
| CpG | C | chr10:129467333 |
| CpG | C | chr10:129467346 |
| CpG | C | chr10:129467351 |
| CpG | C | chr10:129467363 |
| CpG | C | chr10:129467379 |
| CpG | C | chr10:129467390 |
| CpG | C | chr10:129467393 |
| CpG | C | chr10:129467396 |
| CpG | C | chr10:129467408 |
| CpG | C | chr10:129467429 |
| CpG | C | chr10:129467433 |
| CpG | C | chr10:129467446 |

**Library preparation and sequencing**

For each sample, 250 ng of genomic DNA was bisulfite converted using EZ-96 DNA Methylation-Lightning™ Kit (Zymo Research, cat.no. D5033) according to manufacturer’s instructions. Converted DNA was used as a template for *MGMT* gene promoter fragment amplification using KAPA HiFi HotStart Uracil+ ReadyMix PCR Kit (Roche, cat.no. KK2802) and primers that do not overlap with any of the CpG dinucleotides (forward primer sequence: GAT CTA CAC TCT TTC CCT ACA CGA CGC TCT TCC GAT CT agg ttg ggt aat att tgg gag; reverse primer sequence: GTG ACT GGA GTT CAG ACG TGT GCT CTT CCG ATC T ctc tct tac ttt tct caa atc ctc). Betaine was added to every PCR mix at a final concentration of 0.5 M to enhance amplification of GC-rich sequences and thus decrease potential PCR bias towards unmethylated sequences. The following thermal cycling conditions were used: 95 °C for 5 min, then 35 cycles of 98 °C for 20 s, 60 °C for 20 s, and 72 °C for 15 s, before a final elongation step of 72 °C for 1 min. Illumina indexes (UDIs without UMIs) were added to the amplicon mix using xGen™ UDI 10nt Primer Plates 1-8 (IDT, cat.no. 10008053) and KAPA HiFi HotStart Uracil+ ReadyMix PCR Kit (Roche, cat.no. KK2802) with the following thermal cycling conditions: 98 °C for 45 s, then 6 cycles of 98 °C for 15 s, 66 °C for 30 s, and 72 °C for 30 s, before a final elongation step of 72 °C for 1 min. Indexed libraries were purified using Mag-Bind® Total Pure NGS Kit (Omega Bio-Tek, cat.no. M1378-01) with a bead ratio of 0.65x and quantified using Quant-iT™ PicoGreen™ dsDNA Assay Kit (ThermoFisher Scientific, cat.no. P11496). Indexed libraries from 384 samples were pooled together and sequenced using Illumina MiSeq Reagent Kit v3 (Illumina, cat.no. MS-102-3003) on an Illumina MiSeq System (Illumina), aiming to achieve an ultra-deep coverage of more than 30,000x for each amplicon (by 2x226 bp long paired end sequencing reads).

**Mapping, methylation calling and statistical analysis**

Reads were mapped/aligned to the GRCh38 reference genome and the methylation was called using Illumina DRAGEN Bio-IT Platform (v3.9.5) with the following settings: --Aligner.aln-min-score -1000000, --Aligner.min-score-coeff 0, --Aligner.match-score 1, --Aligner.pe-stat-mean-insert 385, --Aligner.pe-stat-stddev-insert 20.0, --Aligner.pe-stat-mean-read-len 220, --Aligner.pe-stat-quartiles-insert "355 385 420". The R software environment for statistical computing (v4.4.0) was used for all downstream statistical analyses. Calling hypermethylated variant epiallele frequencies (VEFs) was performed using epialleleR R package (https://bioconductor.org/packages/epialleleR/, v1.11.2) (8) with the following parameters: min.mapq=30, min.baseq=20, threshold.reads=TRUE, threshold.context="CG", min.context.sites=20, min.context.beta=0.5, max.outofcontext.beta=0.1. Allele frequencies of SNP rs16906252 in methylated and unmethylated reads were determined using function epialleleR::generateVcfReport. Methylation patterns were extracted from alignment data using function epialleleR::extractPatterns. Sequencing coverage for all samples is detailed in Additional file 5.

*MGMT* promoter regions, where epimutations were to be assessed, were defined blinded to case-control status of samples. Preliminary data for the regions are further described in the study protocol amendment (Additional file 2). According to the study protocol, for each of the regions A-C (Supplementary Figure S1), an epimutation was defined as a methylation pattern per allele with at least 75% of CpGs being informative, and more than a half of informative ones being methylated, and an epimutation-positive sample was defined as a sample with the coverage of at least 10000x and an epimutation frequency (Variant Epiallele Frequency, VEF) of at least 1/10000.

Primary analysis assessed epimutation frequency (VEF) in the region B, overlapping *MGMT* exon 1, while secondary analyses assessed regions A and C as well as entire PCR amplicon region. Hazard ratio (HR) estimation was performed using Cox proportional hazards regression in matching case-control groups, with the model including age, race/ethnicity, previous hormone use, smoking and DNA extraction method as independent variables (covariates). Subgroups were stratified by rs16906252 genotype when assessing cancer risk for epimutation carriers, or by methylation status when assessing cancer risk for carriers of alternative rs16906252 SNP allele.

**Supplementary Results**

**Assay characteristics**

**Supplementary Figure S2. Average depth of coverage and methylation of *MGMT* promoter**


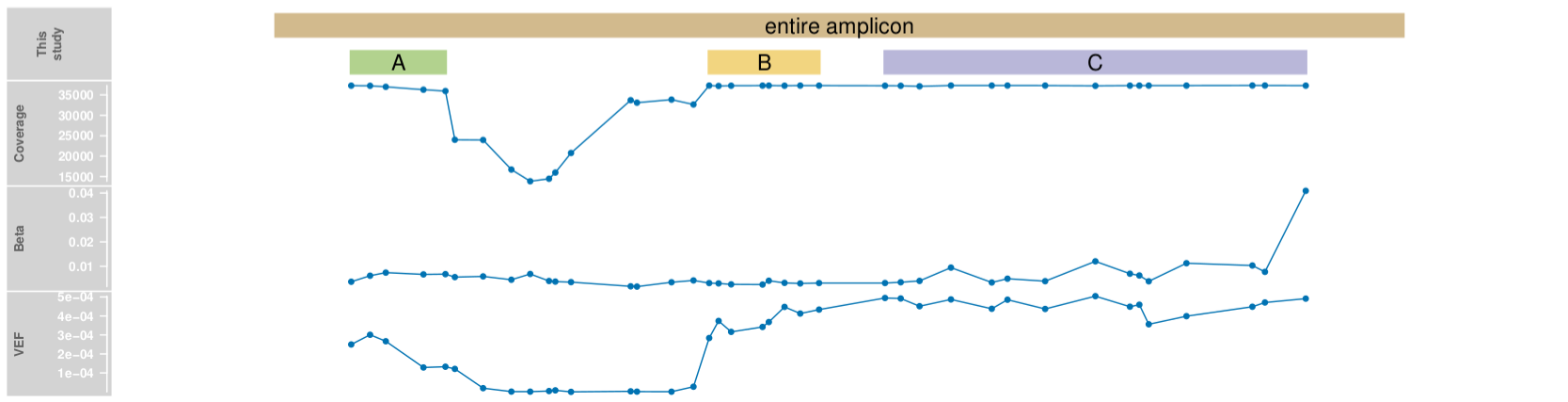
Assessed *MGMT* promoter regions (top), as well as average per-sample coverage, average methylation beta values and average frequencies of epimutation (VEF values) indicated as blue lines (dots represent single CpGs). Per-sample coverage is further detailed in Additional file 5.

**Supplementary Figure S3. Observed versus expected methylation levels in control samples.**


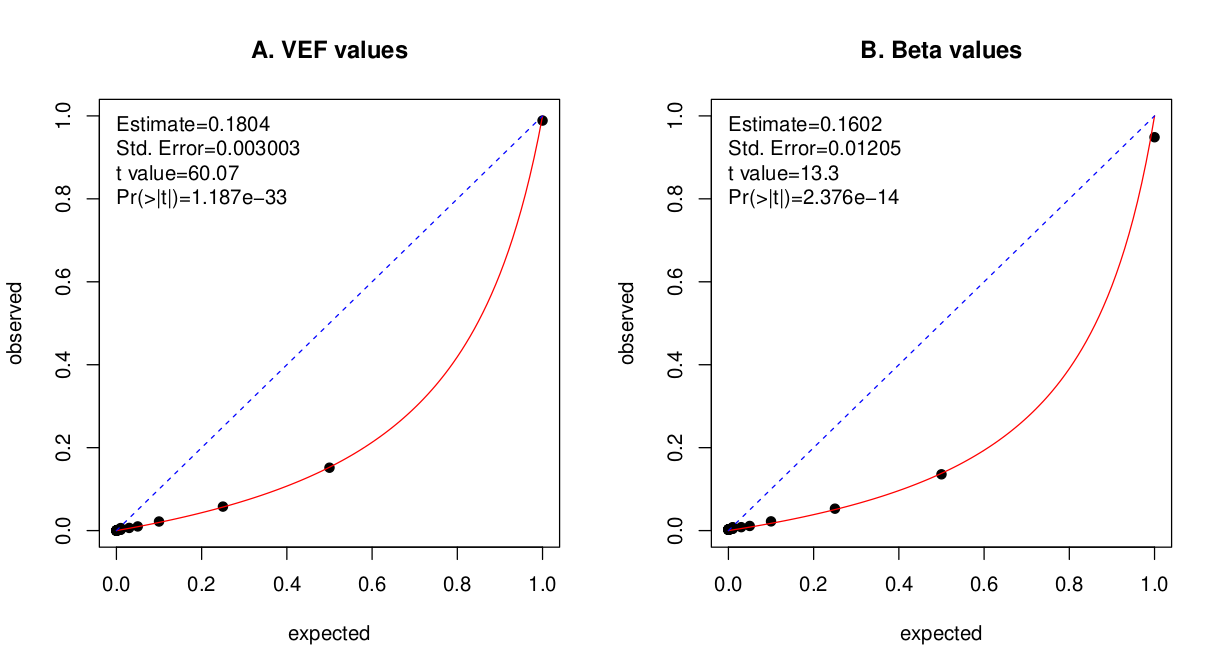
Observed versus expected methylation levels in a dilution series of fully methylated DNA in unmethylated DNA. Methylation calculated as A) VEF and B) beta values, averaged for the entire assessed promoter area. Dotted blue line indicates optimal concordance between observed and expected results. Black dots (with solid red trend line) indicate the results from the analysed dilution series.

**Supplementary Figure S4. Distribution of methylation beta values as compared to VEF values**


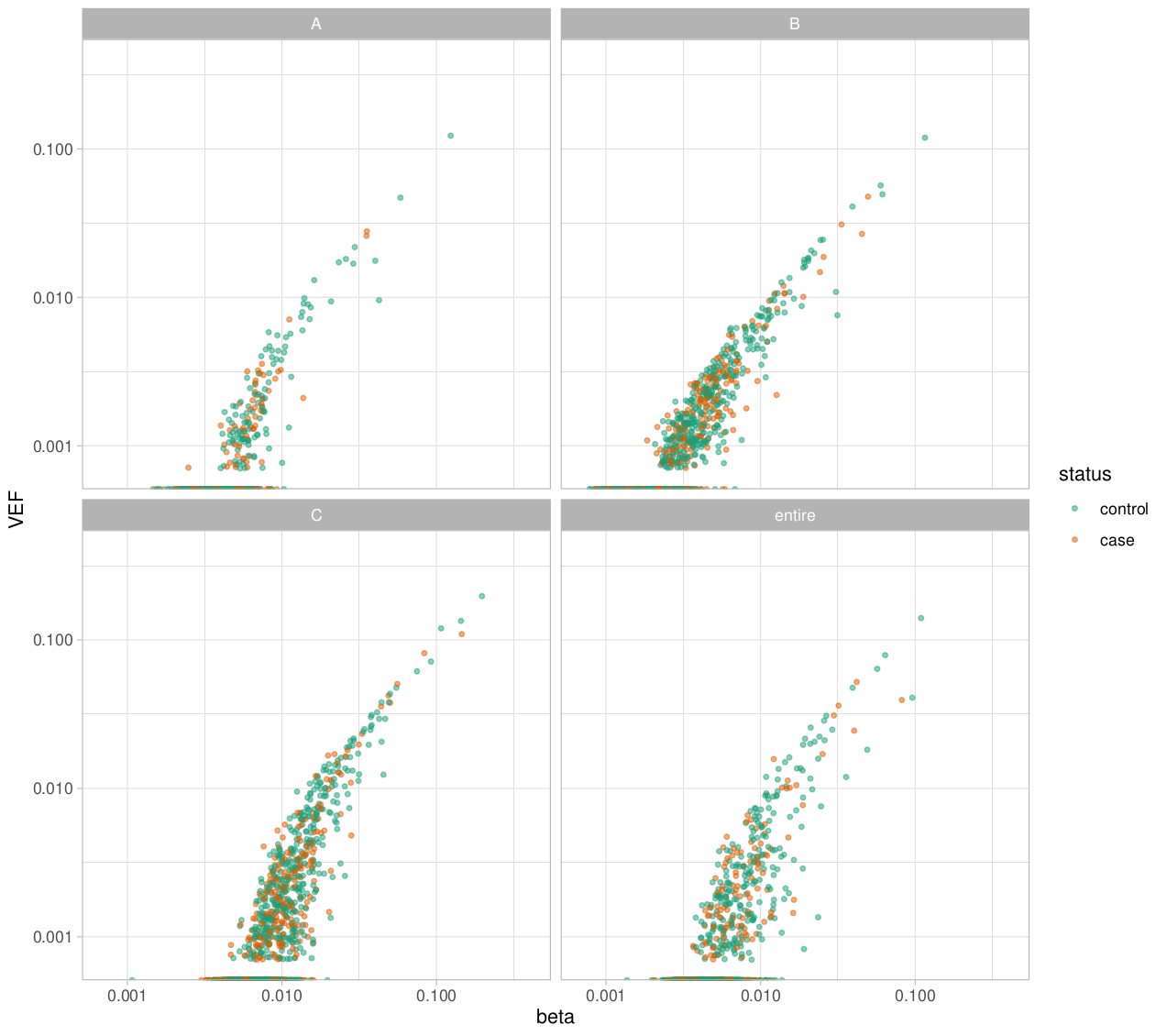
Scatter plot showing the relationship between methylation beta (x-axis) and VEF (y-axis) values for all samples (cases and controls) analyzed in the present study.

**Supplementary Figure S5. Histogram of VEF values for the assessed *MGMT* promoter regions**


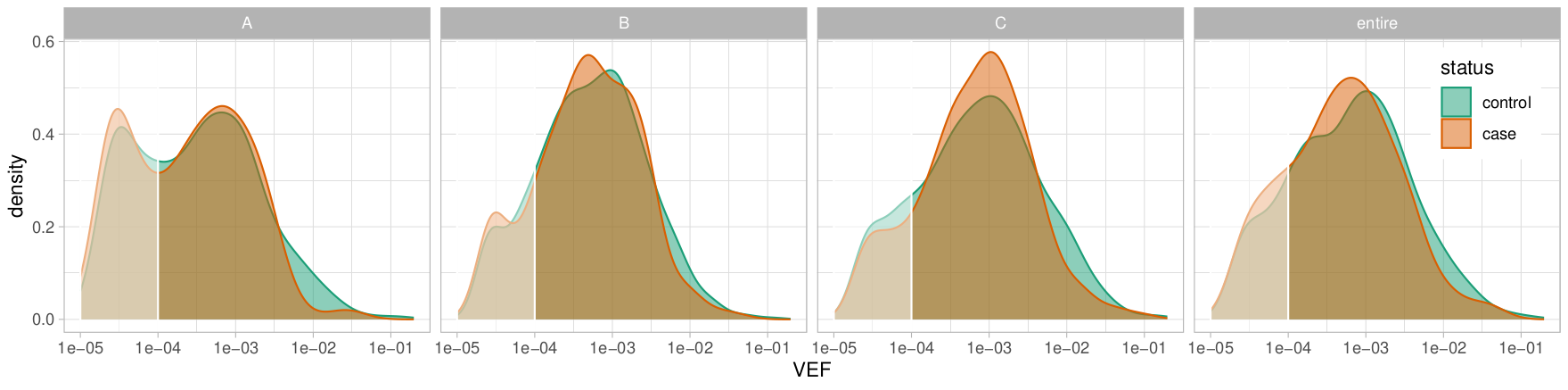
Probability density plots of VEF values for all samples (cases and controls) analyzed in the present study, split by the assessed regions of *MGMT* promoter. The VEF cutoff value (equals 1e-4) for samples scored as methylation positive, applied for all main analyses, is represented by a vertical white line.

**Supplementary Figure S6. Histogram of VEF values for GB cases and controls by region and rs16906252 genotype**


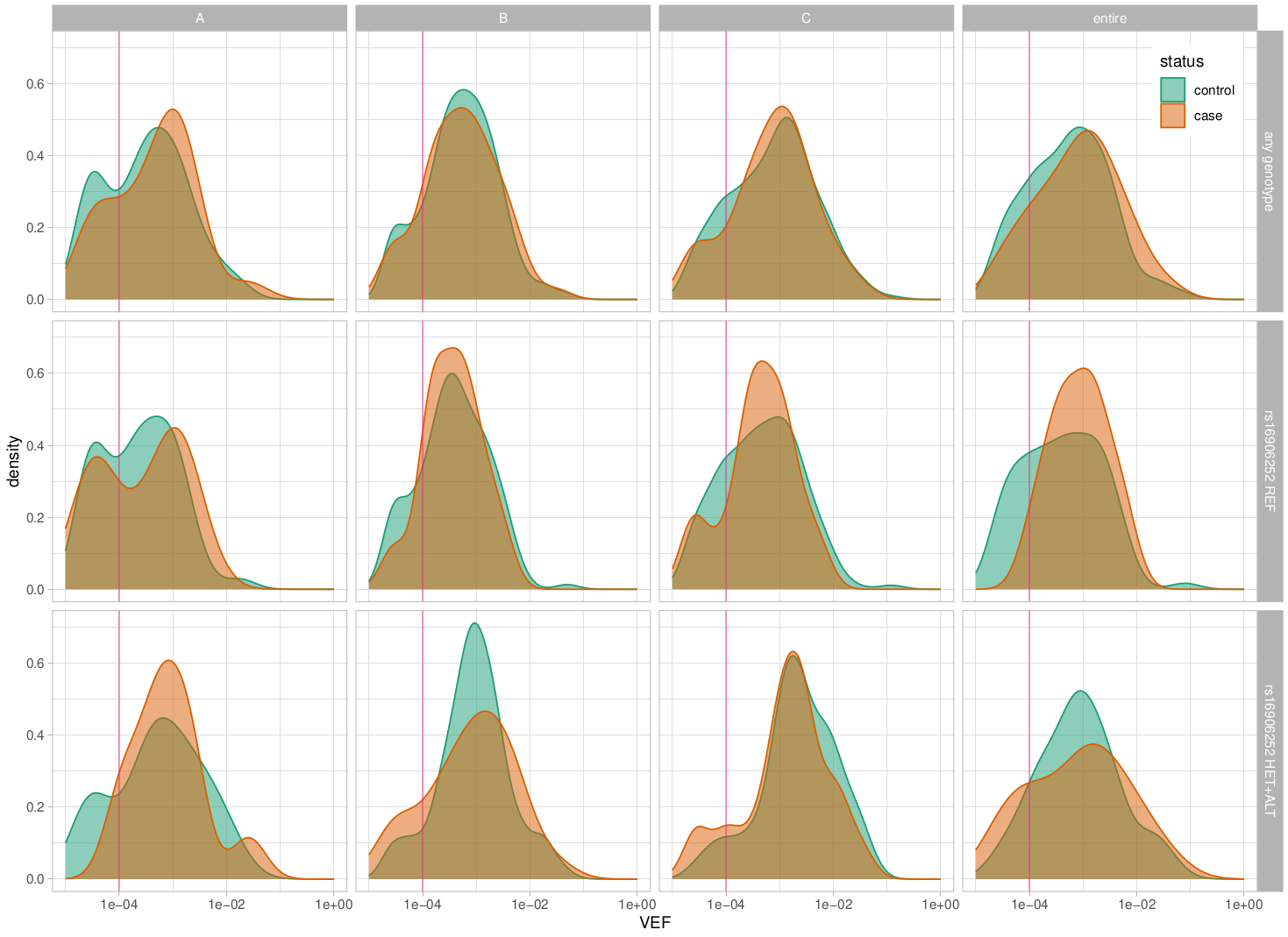
Probability density plots of VEF values for GB cases and controls, split by assessed region of *MGMT* promoter and genotype of SNP rs16906252. The VEF cutoff value (equals 1e-4) applied for all main analyses is represented by a vertical pink line.

**Supplementary Figure S7. Histogram of VEF values for DLBCL cases and controls by region and rs16906252 genotype**


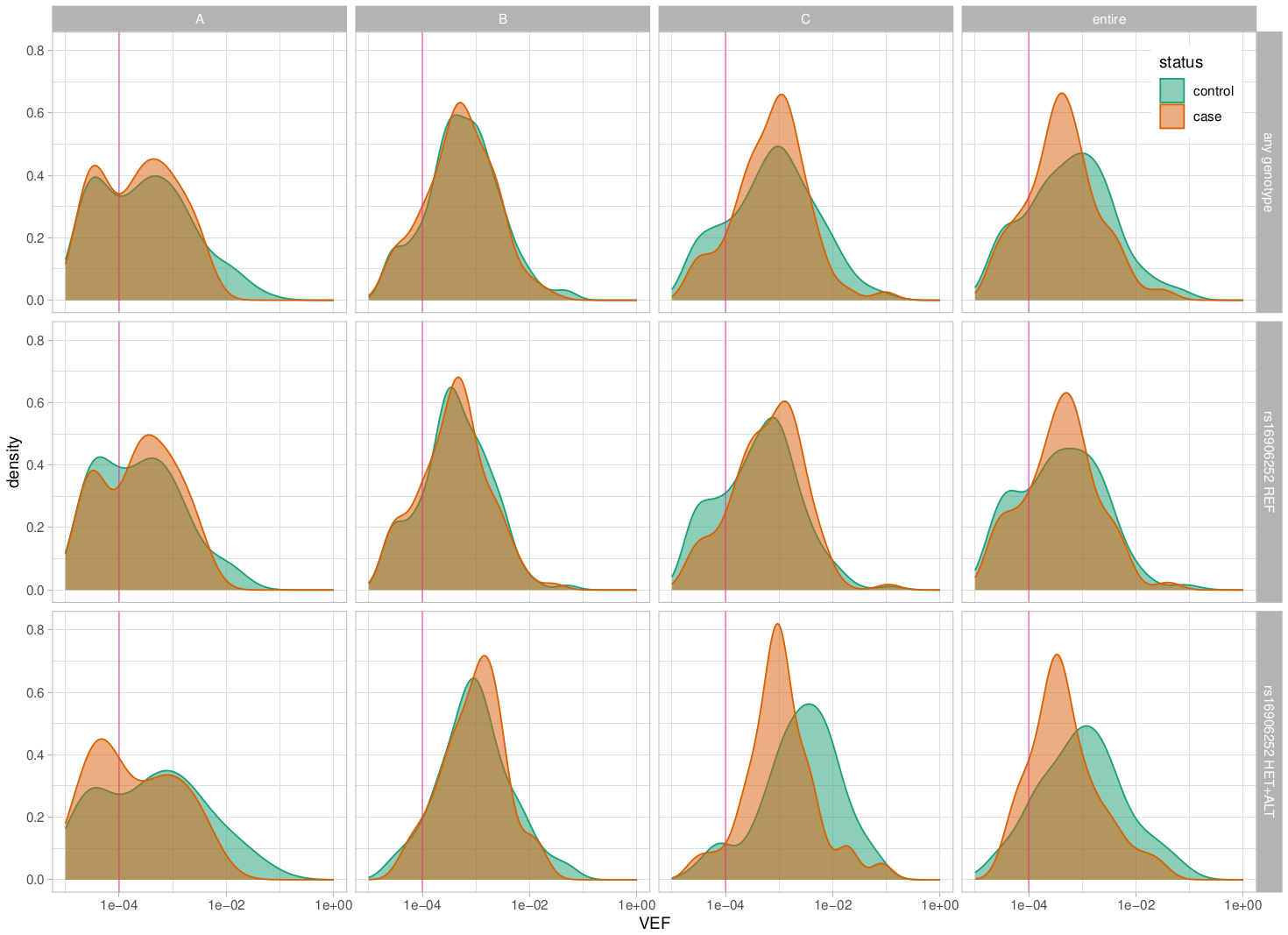
Probability density plots of VEF values for DLBCL cases and controls, split by assessed region of *MGMT* promoter and genotype of SNP rs16906252. The VEF cutoff value (equals 1e-4) applied for all main analyses is represented by a vertical pink line.

**Supplementary Figure S8. Histogram of VEF values for all CC cases and controls by region and rs16906252 genotype**


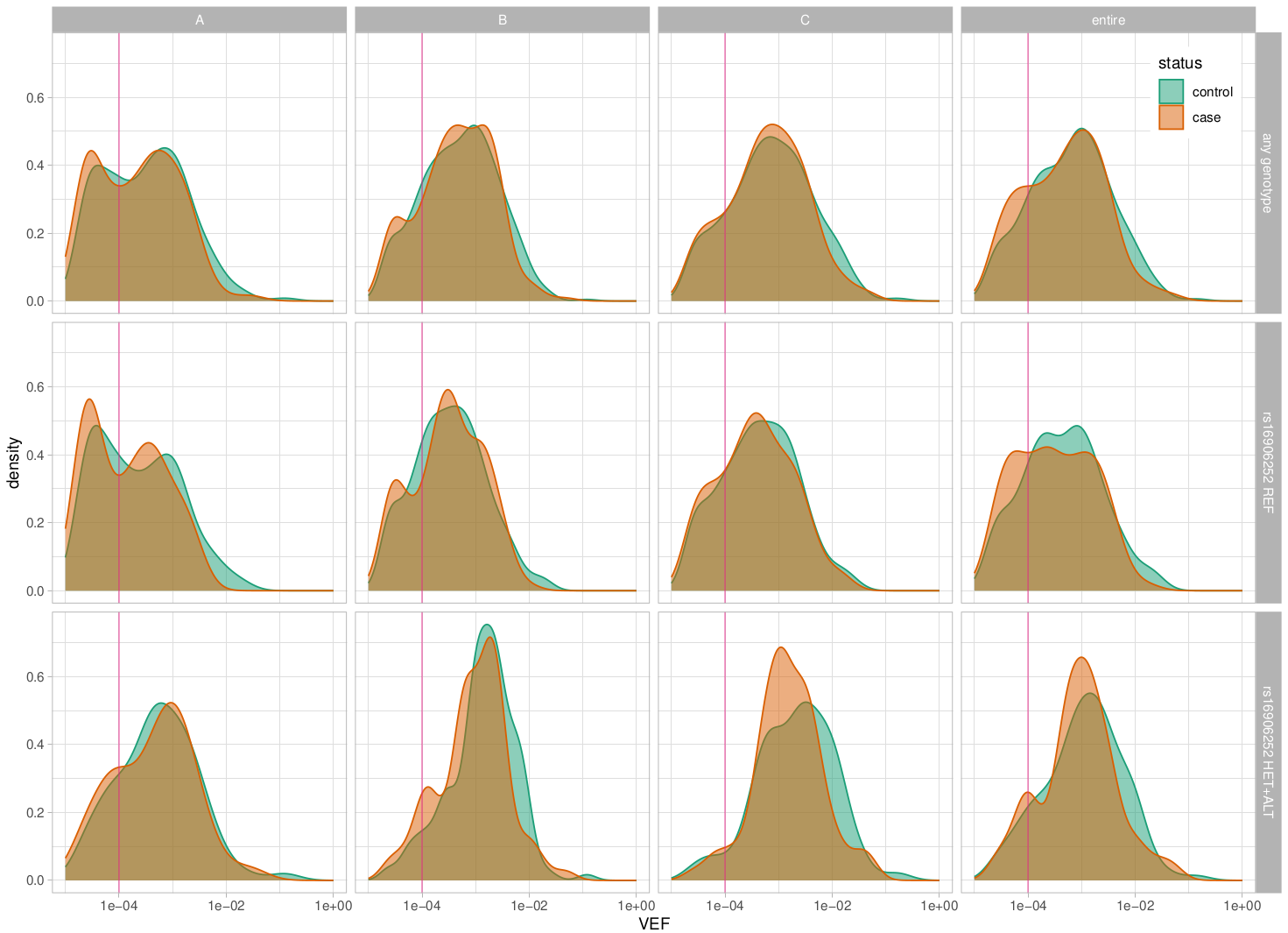
Probability density plots of VEF values for CC cases and controls, split by assessed region of *MGMT* promoter and genotype of SNP rs16906252. The VEF cutoff value (equals 1e-4) applied for all main analyses is represented by a vertical pink line.

**Supplementary Figure S9. Histogram of VEF values for left-sided CC cases and controls by region and rs16906252 genotype**


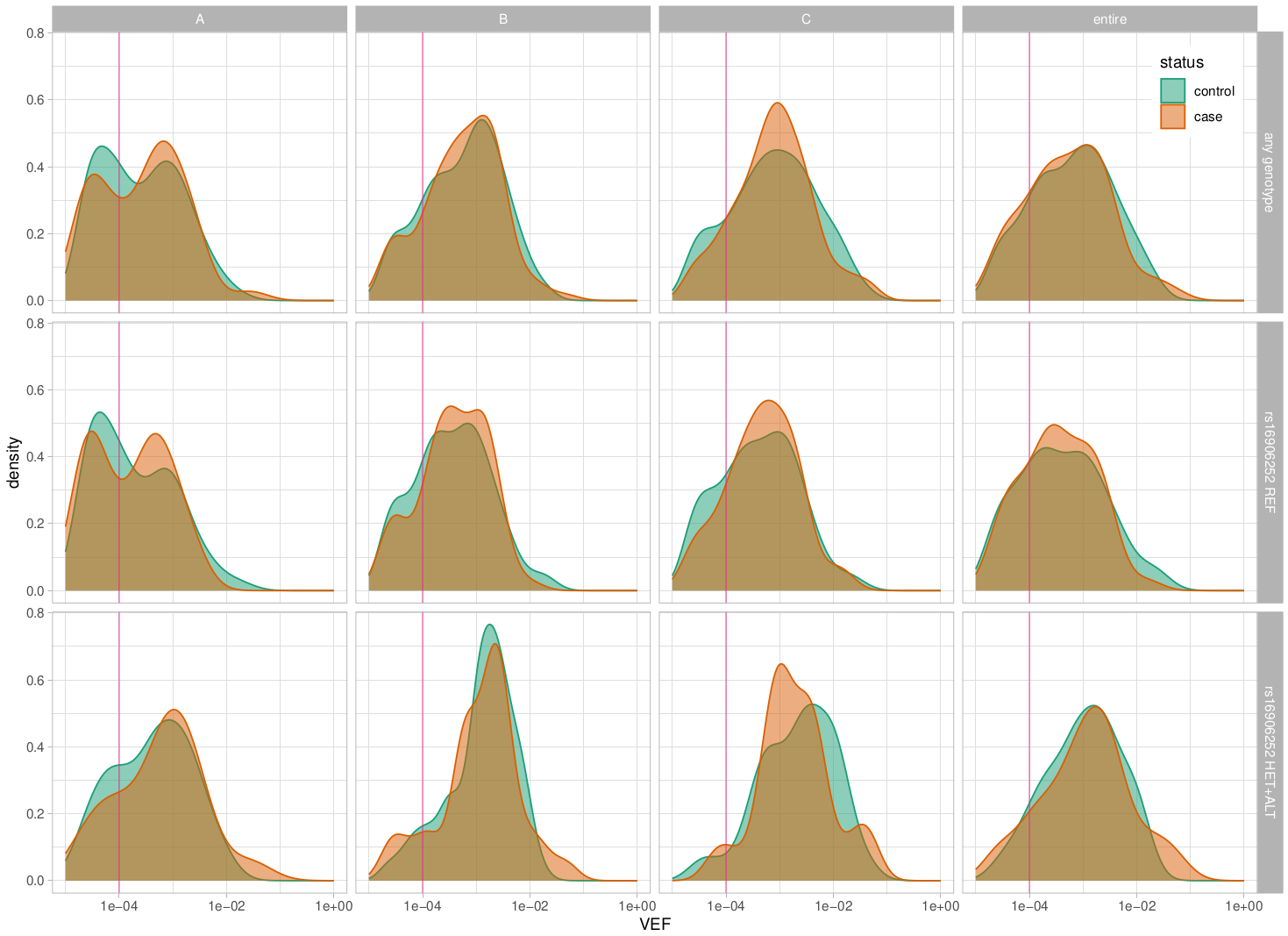
Probability density plots of VEF values for left-sided CC cases and controls, split by assessed region of *MGMT* promoter and genotype of SNP rs16906252. The VEF cutoff value (equals 1e-4) applied for all main analyses is represented by a vertical pink line.

**Supplementary Figure S10. Histogram of VEF values for right-sided CC cases and controls by region and rs16906252 genotype**


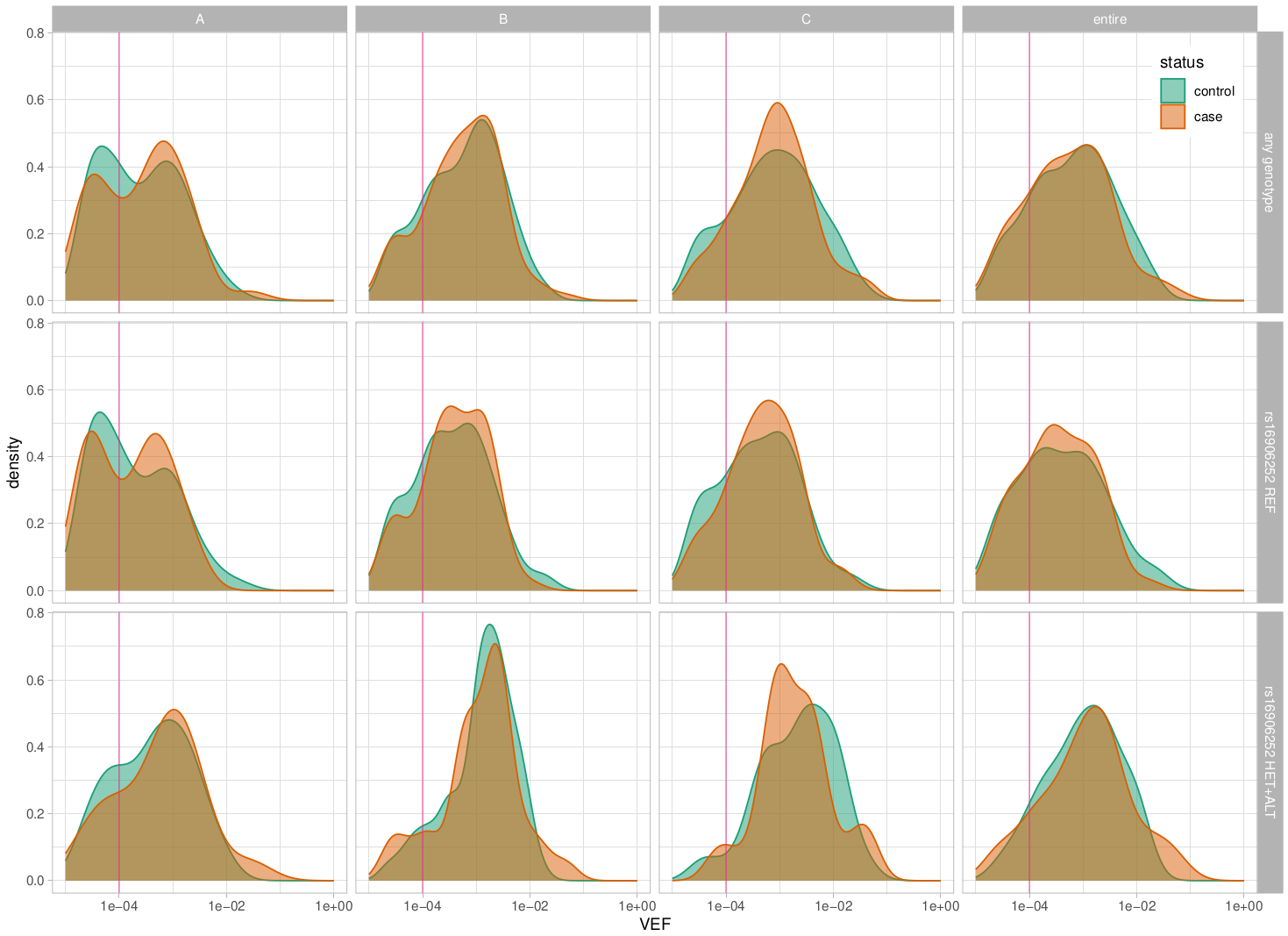
Probability density plots of VEF values for right-sided CC cases and controls, split by assessed region of *MGMT* promoter and genotype of SNP rs16906252. The VEF cutoff value (equals 1e-4) applied for all main analyses is represented by a vertical pink line.

**Risk estimates for subregions of the *MGMT* promoter area**

**Supplementary Figure S11. Risk for glioblastoma by region and rs16906252 genotype**


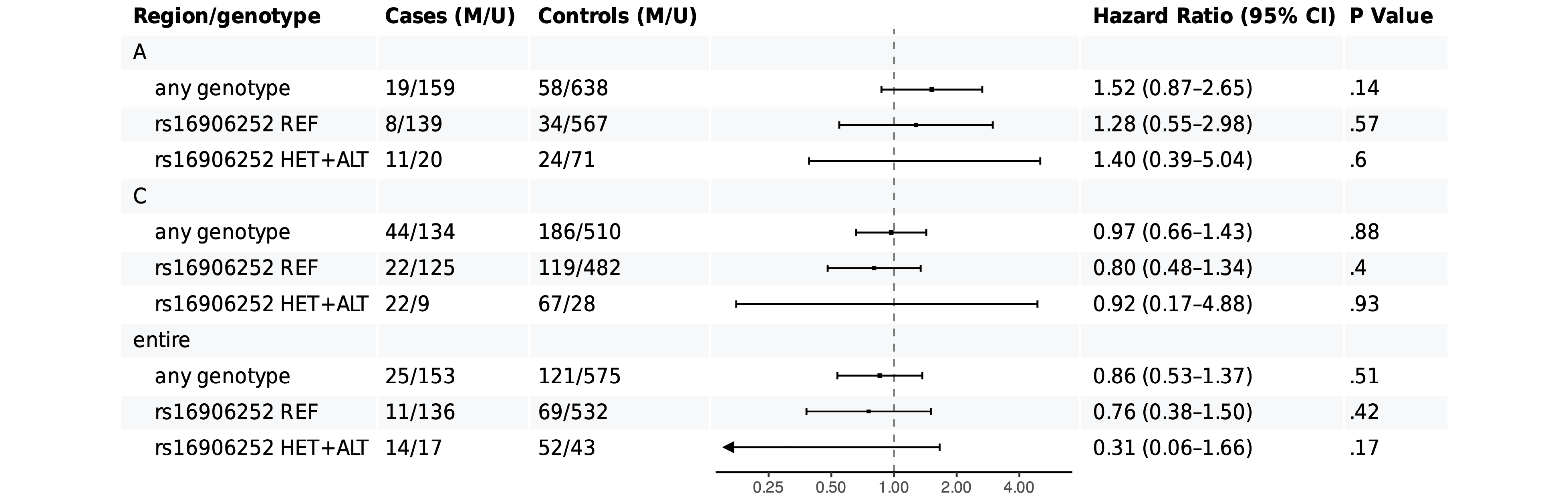
Risk of incident glioblastoma according to methylation in regions A, C, or entire assayed promoter area (Supplementary Figure S1), split by rs16906252 genotype.

**Supplementary Figure S12. Risk for DLBCL by region and rs16906252 genotype**


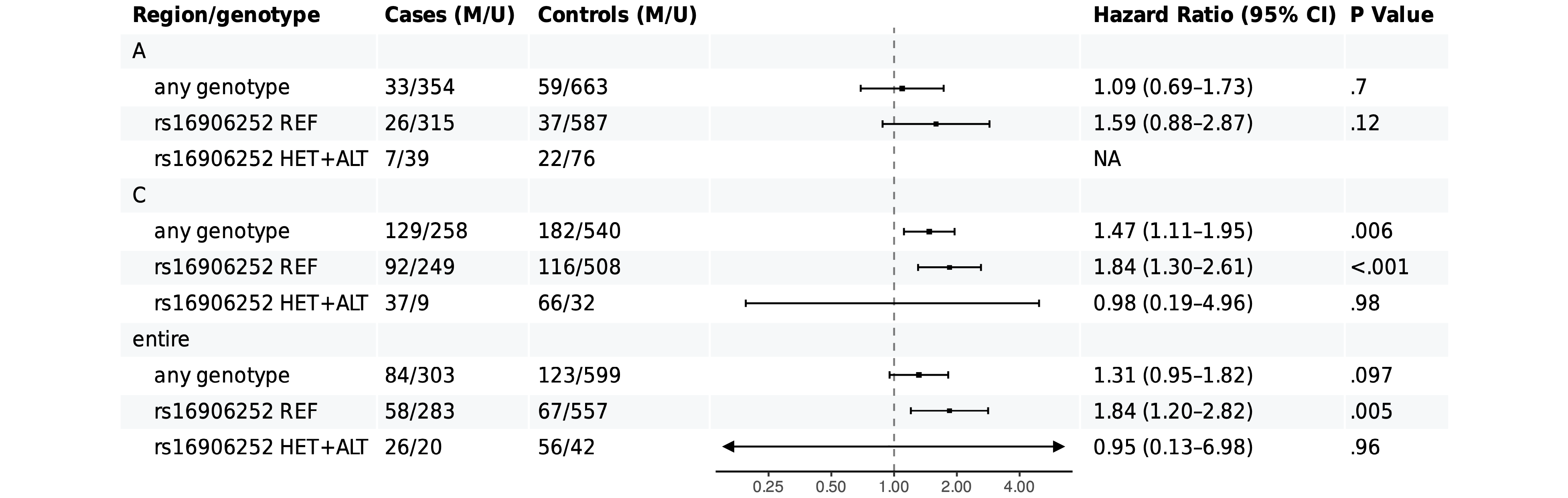
Risk of incident DLBCL according to methylation in regions A, C, or entire assayed promoter area (Supplementary Figure S1), split by rs16906252 genotype.

**Supplementary Figure S13. Risk for left-sided colon cancer by region and rs16906252 genotype**


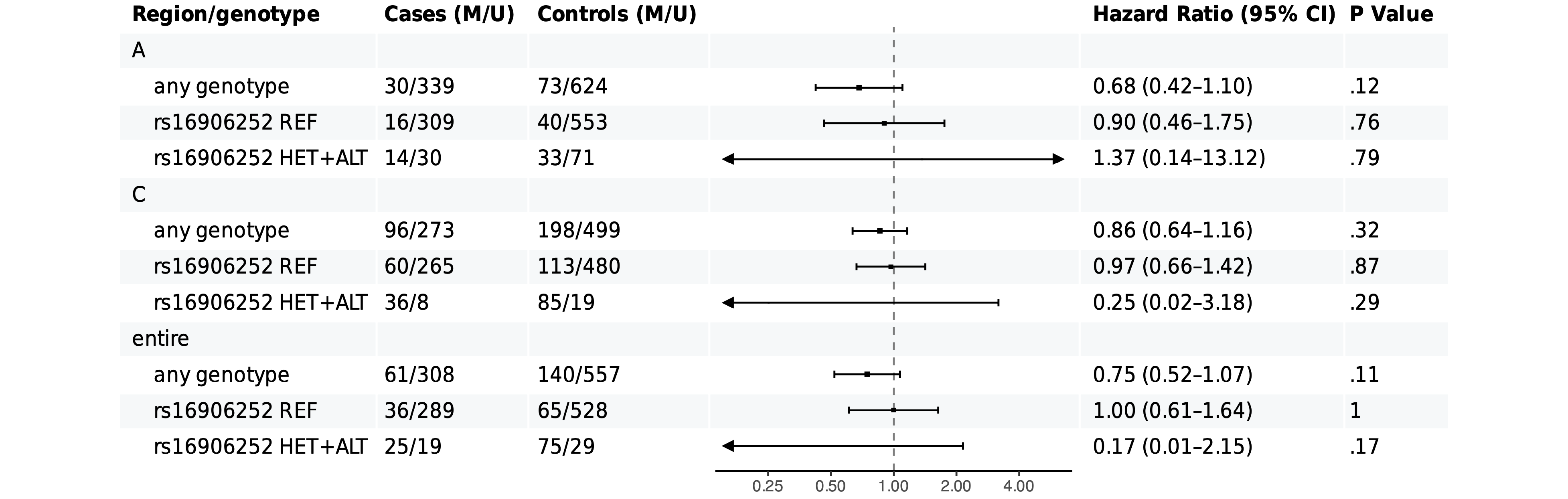
Risk of incident left-sided colon cancer according to methylation in regions A, C, or entire assayed promoter area (Supplementary Figure S1), split by rs16906252 genotype.

**Supplementary Figure S14. Risk for right-sided colon cancer by region and rs16906252 genotype**


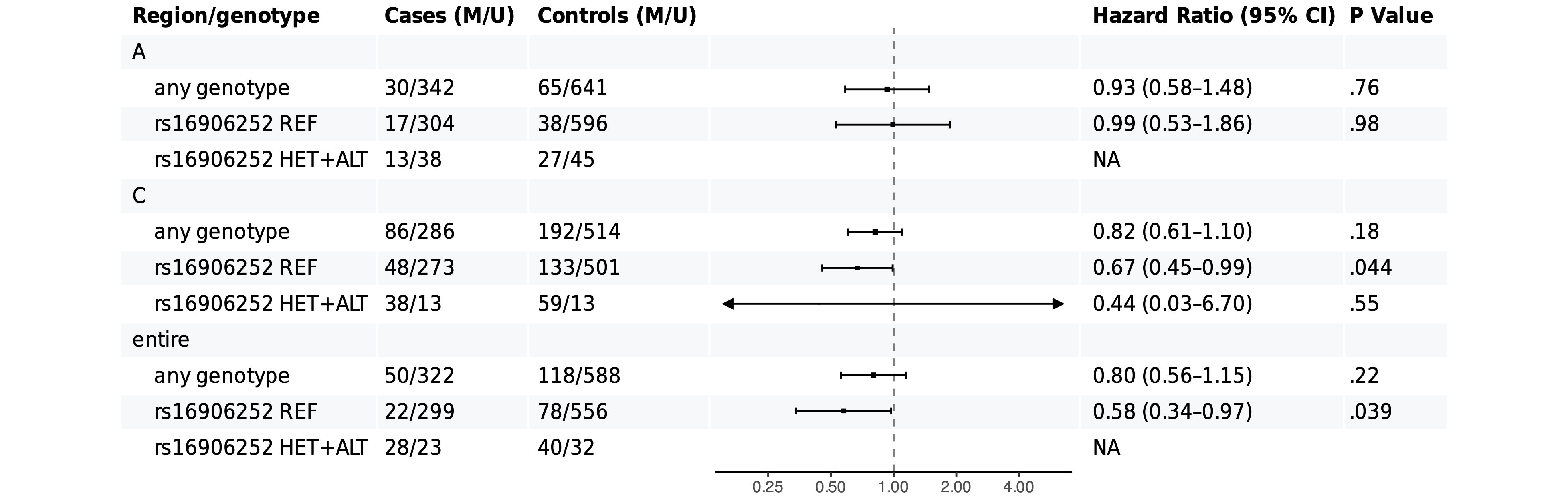
Risk of incident right-sided colon cancer according to methylation in regions A, C, or entire assayed promoter area (Supplementary Figure S1), split by rs16906252 genotype.

**Supplementary Figure S15. Risk for colon cancer by region and rs16906252 genotype**


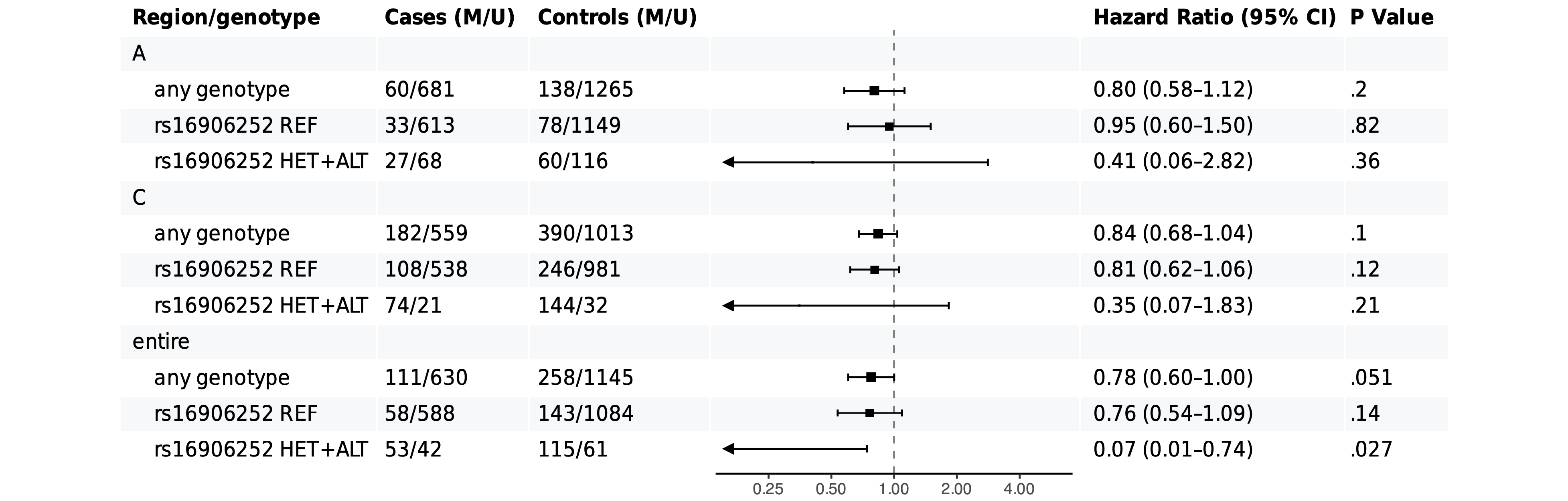
Risk of incident colon cancer according to methylation in regions A, C, or entire assayed promoter area (Supplementary Figure S1), split by rs16906252 genotype.

**Supplementary Table S2. Frequency of rs16906252 genotypes in cases and controls**

| **category** | | **rs16906252 genotype** | | |
| --- | --- | --- | --- | --- |
|  |  | **REF** | **HET** | **ALT** |
| All cases | | 1126 (86.8%) | 170 (13.1%) | 1 (0.08%) |
| All controls | | 2076 (87.1%) | 293 (12.3%) | 14 (0.6%) |
| Glioblastoma | cases | 147 (82.6%) | 31 (17.4%) | 0 (0%) |
|  | controls | 601 (86.4%) | 92 (13.2%) | 3 (0.4%) |
| DLBCL | cases | 341 (88.1%) | 46 (11.9%) | 0 (0%) |
|  | controls | 624 (86.4%) | 95 (13.2%) | 3 (0.4%) |
| Left-sided colon cancer | cases | 325 (88.1%) | 43 (11.7%) | 1 (0.3%) |
|  | controls | 593 (85.1%) | 100 (14.3%) | 4 (0.6%) |
| Right-sided colon cancer | cases | 321 (86.3%) | 51 (13.7%) | 0 (0%) |
|  | controls | 634 (89.8%) | 65 (9.2%) | 7 (1.0%) |

**Supplementary Table S3. Frequency of rs16906252 genotypes in methylation-positive and negative samples**

| **Subregion methylation** | | **rs16906252 genotype** | | | **Odds ratio**  **[95% CI]** | **P Value** |
| --- | --- | --- | --- | --- | --- | --- |
|  |  | **REF** | **HET** | **ALT** |  |  |
| A | methylated | 212 (58.2%) | 144 (39.6%) | 8 (2.2%) | 6.42  [5.03–8.17] | <.001 |
|  | unmethylated | 3172 (90.0%) | 343 (9.7%) | 11 (0.3%) |  |  |
| B | methylated | 658 (63.0%) | 372 (35.6%) | 15 (1.4%) | 13.5  [10.7–17.0] | <.001 |
|  | unmethylated | 2726 (95.8%) | 115 (4.0%) | 4 (0.1%) |  |  |
| C | methylated | 661 (63.0%) | 372 (35.5%) | 16 (1.5%) | 13.5  [10.8–17.1] | <.001 |
|  | unmethylated | 2723 (95.8%) | 115 (4.0%) | 3 (0.1%) |  |  |
| Entire amplicon | methylated | 385 (56.5%) | 285 (41.9%) | 11 (1.6%) | 11.0  [8. 9–13.6] | <.001 |
|  | unmethylated | 2999 (93.5%) | 202 (6.3%) | 8 (0.2%) |  |  |

Odds ratios and Fisher’s exact test P values were calculated assuming dominant genetic model (REF versus HET+ALT combined).

**Risk estimates for rs16906252 variant alleles**

**Supplementary Figure S16. Risk for glioblastoma by promoter region and its methylation status**


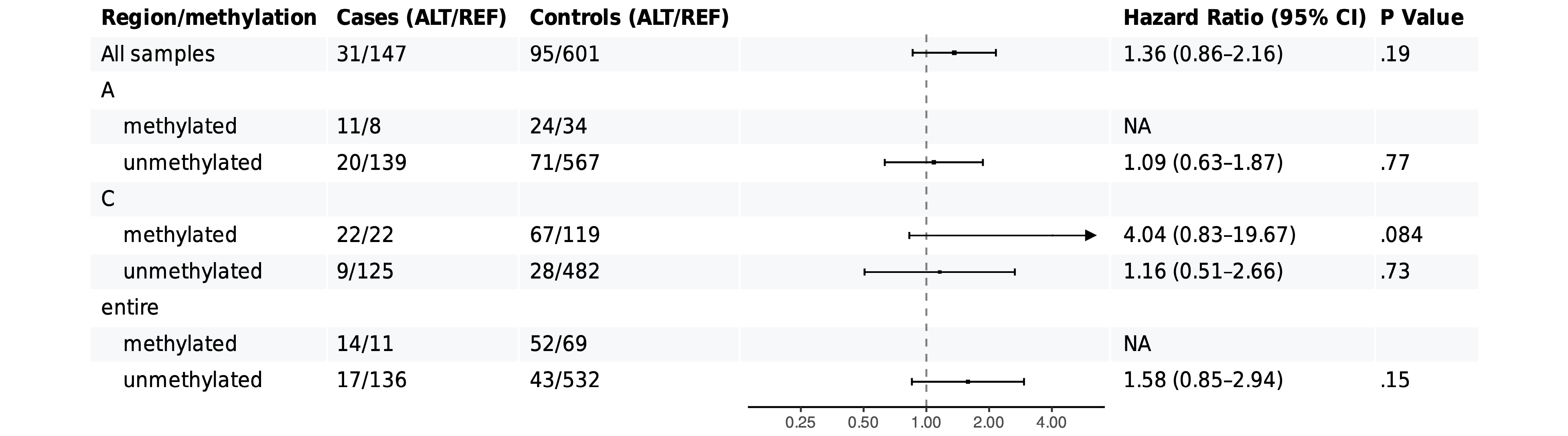
 Risk of incident glioblastoma for rs16906252 variant alleles, according to methylation in regions A, C, or entire assayed promoter area (Supplementary Figure S1).

**Supplementary Figure S17. Risk for DLBCL by promoter region and its methylation status**


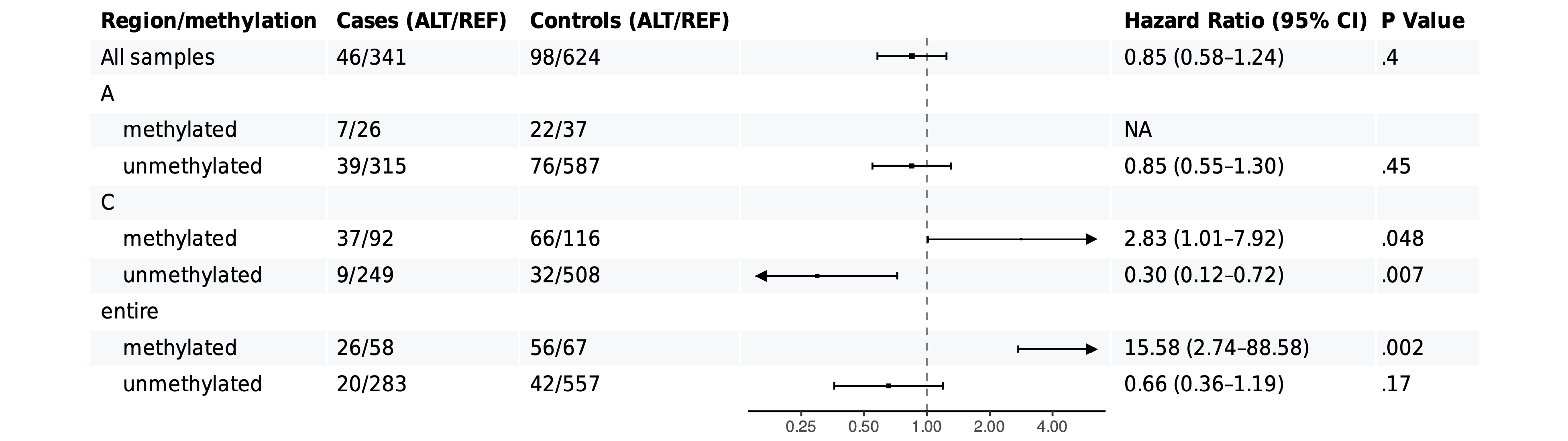
 Risk of incident DLBCL for rs16906252 variant alleles, according to methylation in regions A, C, or entire assayed promoter area (Supplementary Figure S1).

**Supplementary Figure S18. Risk for left-sided colon cancer by promoter region and its methylation status**


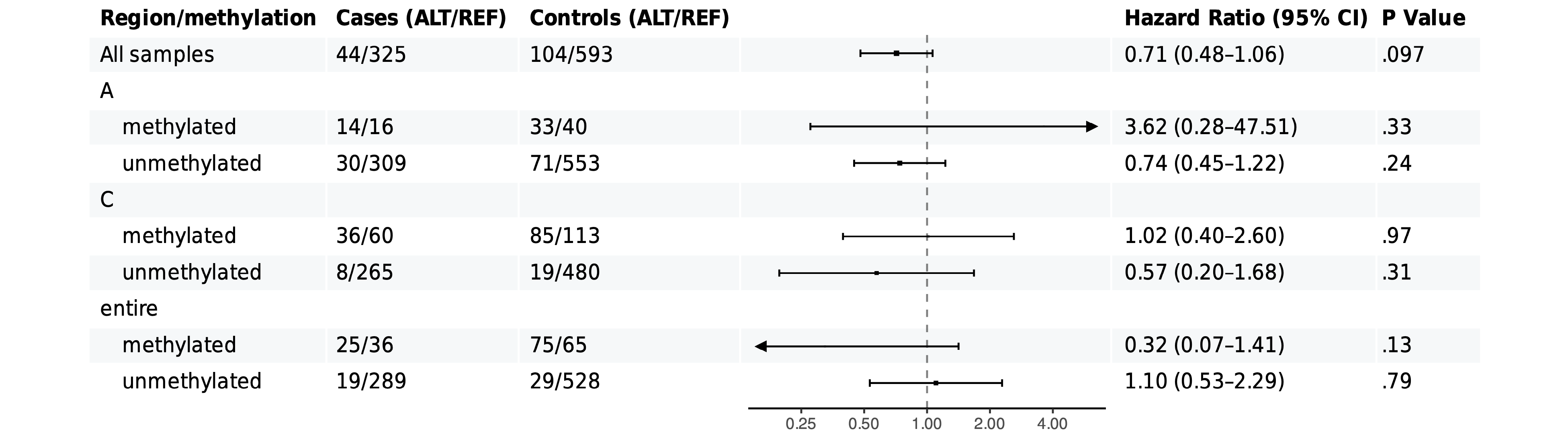
 Risk of incident left-sided colon cancer for rs16906252 variant alleles, according to methylation in regions A, C, or entire assayed promoter area (Supplementary Figure S1).

**Supplementary Figure S19. Risk for right-sided colon cancer by promoter region and its methylation status**


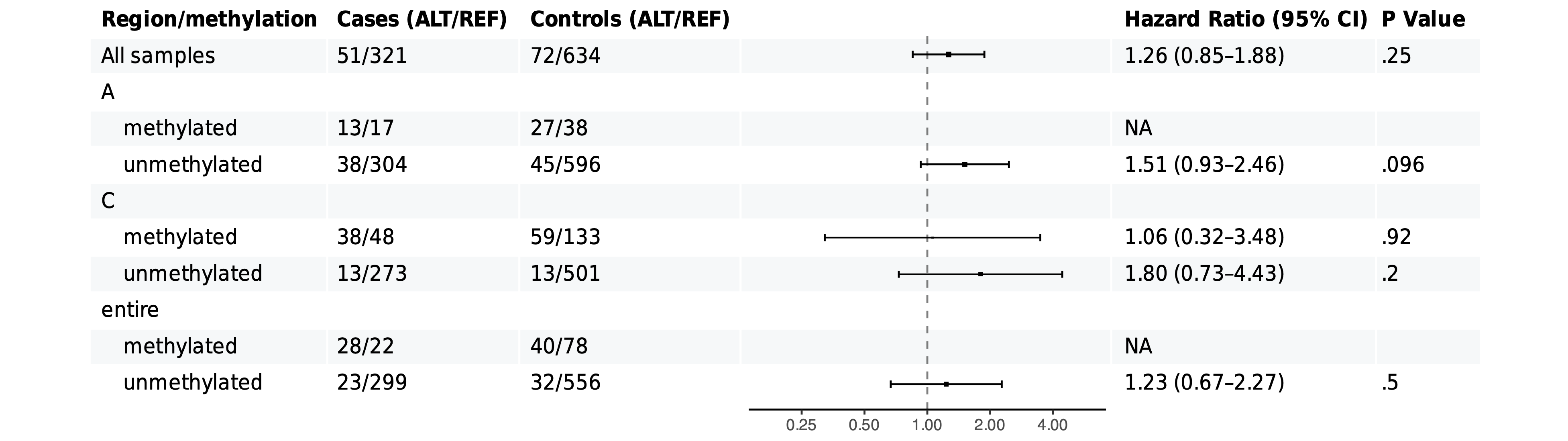
 Risk of incident right-sided colon cancer for rs16906252 variant alleles, according to methylation in regions A, C, or entire assayed promoter area (Supplementary Figure S1).

**Supplementary Figure S20. Risk for colon cancer by promoter region and its methylation status**


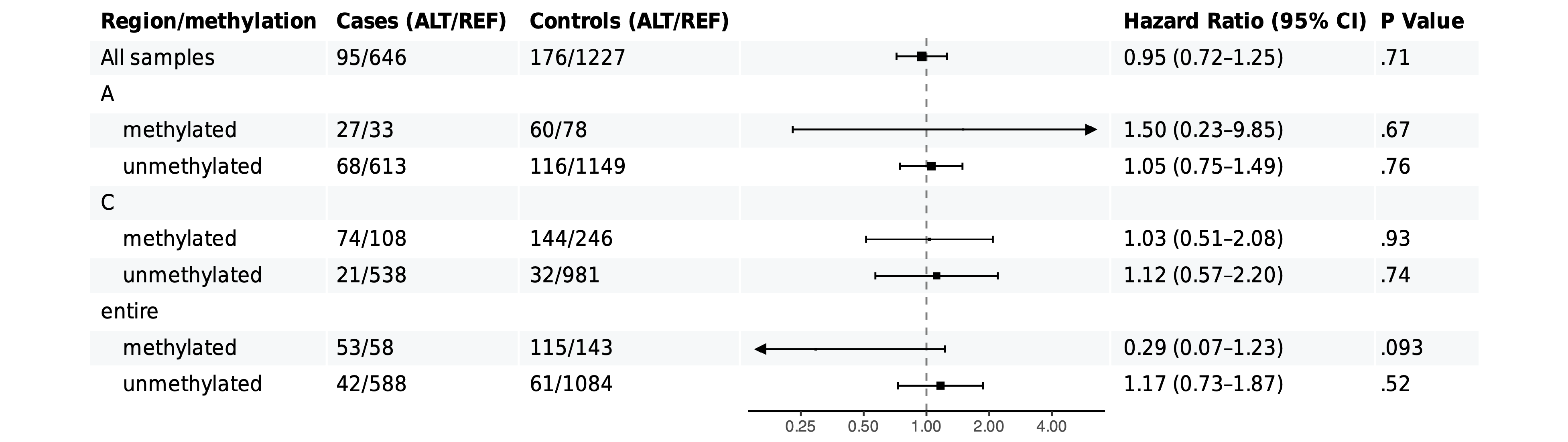
 Risk of incident colon cancer for rs16906252 variant alleles, according to methylation in regions A, C, or entire assayed promoter area (Supplementary Figure S1).

**Allele specificity of methylation**

**Supplementary Figure S21. Preferential methylation of ALT-allele of rs16906252**

(A) (B)


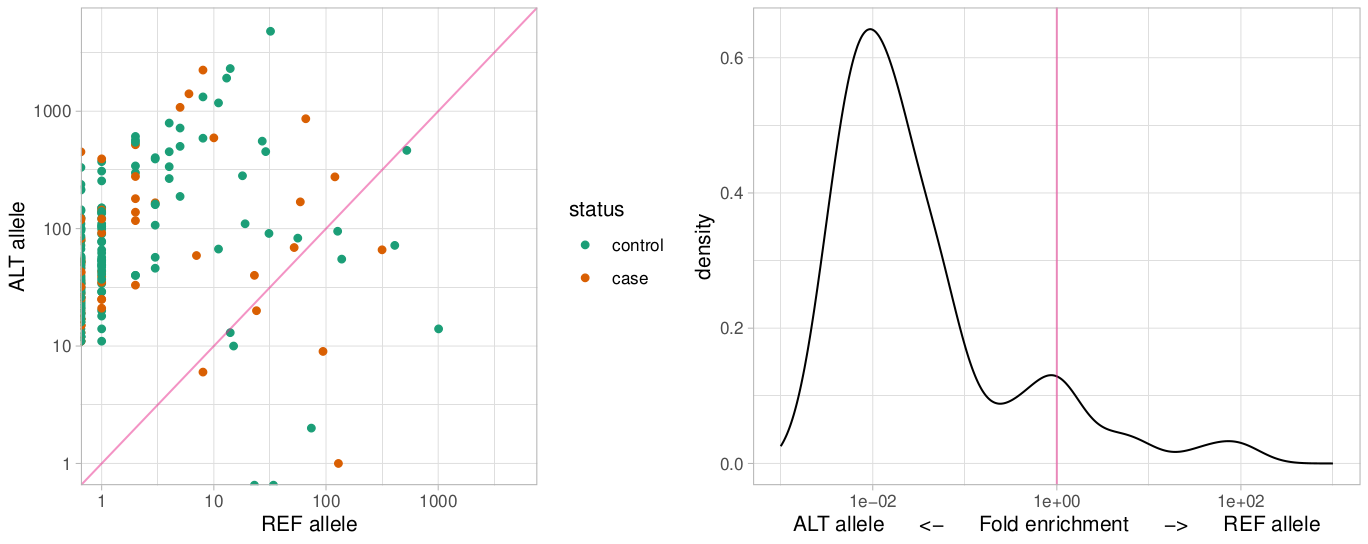
(A) Counts of methylated reads carrying reference (x-axis) or alternative (y-axis) allele of the SNP rs16906252 for all *MGMT*-methylated samples heterozygous for rs16906252 (n=230). (B) Density distribution of allele-specific methylation assessed as fold enrichment (FE). FE for association between SNP allele and methylation status was calculated as FE=(REF^meth^*(ALT^meth^+ALT^unmeth^))/((REF^meth^+REF^unmeth^)*ALT^meth^) for all heterozygous methylated samples, where X^meth^ and X^unmeth^ are the number of methylated or unmethylated reads, carrying reference (REF) or alternative (ALT) SNP allele, respectively. FE>1 if reference SNP allele occurs more often within methylated strands.

**Supplementary References**

1. Esteller M, Hamilton SR, Burger PC, Baylin SB, Herman JG: Inactivation of the DNA repair gene O6-methylguanine-DNA methyltransferase by promoter hypermethylation is a common event in primary human neoplasia. Cancer Res 1999, 59:793-797.

2. Estival A, Sanz C, Ramirez JL, Velarde JM, Domenech M, Carrato C, de las Penas R, Gil-Gil M, Sepulveda J, Armengol R, et al: Pyrosequencing versus methylation-specific PCR for assessment of MGMT methylation in tumor and blood samples of glioblastoma patients. Scientific Reports 2019, 9.

3. Brawanski KR, Sprung S, Freyschlag CF, Hoeftberger R, Strobel T, Haybaeck J, Thome C, Manzl

C, Birkl-Toeglhofer AM: Influence of MMR, MGMT Promotor Methylation and Protein Expression on Overall and Progression-Free Survival in Primary Glioblastoma Patients Treated with Temozolomide. International Journal of Molecular Sciences 2023, 24.

4. Jensen GL, Pourfarrokh N, Volz M, Morales LL, Walker K, Hammonds KP, El-Ghamry M, Wong LC, Hodjat P, Castro E, Rao A, Jhavar SG: Improved Pathologic response to chemoradiation in MGMT methylated locally advanced rectal cancer. Clinical and Translational Radiation Oncology 2023, 42.

5. Jimenez VG, Doval MB, Bellvert CG, Goliney VG, Asencio OS, Martin AG, Dominguez JI: Quantitative analysis of MGMT promoter methylation status changes by pyrosequencing in recurrent glioblastoma. Neuropathology 2023.

6. Watts GS, Pieper RO, Costello JF, Peng YM, Dalton WS, Futscher BW: Methylation of discrete regions of the O-6-methylguanine DNA methyltransferase (MGMT) CpG island is associated with heterochromatinization of the MGMT transcription start site and silencing of the gene. Molecular and Cellular Biology 1997, 17:5612-5619.

7. Qian XLC, Brent TP: Methylation hot spots in the 5' flanking region denote silencing of the O-6-methylguanine-DNA methyltransferase gene. Cancer Research 1997, 57:3672-3677.

8. Nikolaienko O, Lønning PE, Knappskog S. epialleleR: an R/Bioconductor package for sensitive allele-specific methylation analysis in NGS data. GigaScience 2023;12:giad087.
